# Effectiveness of eHealth for Medication Adherence in Renal Transplant Recipients: Systematic Review and Meta-Analysis

**DOI:** 10.2196/73520

**Published:** 2025-05-13

**Authors:** Lili Zhou, Ke Cheng, Linbin Chen, Xinyi Hou, Jingjing Wan

**Affiliations:** 1 Xiangya College of Nursing Central South University Changsha China; 2 Department of Transplantation Xiangya Third Hospital Central South University Changsha China; 3 Nursing Department Outpatient and Emergency Operating Room The Third Xiangya Hospital of Central South University Changsha China

**Keywords:** kidney transplantation, patient compliance, immunosuppressive therapy, telemedicine, digital interventions, mHealth, tacrolimus, graft survival, artificial intelligence, AI, PRISMA

## Abstract

**Background:**

As the optimal treatment for end-stage renal disease, kidney transplantation has proven instrumental in enhancing patient survival and quality of life. Suboptimal medication adherence is recognized as an independent risk factor for poor prognosis, graft rejection, and graft loss. In recent years, the advancement of IT has facilitated the integration of eHealth technologies into medical medication management, offering potential solutions to improve patient adherence. However, their efficacy in kidney transplant recipients remains inconclusive.

**Objective:**

This study aimed to evaluate the effectiveness of eHealth interventions in improving medication adherence among kidney transplant recipients and identify potential influencing factors.

**Methods:**

We systematically searched PubMed, Web of Science, Cochrane Library, Embase, CINAHL, Scopus, and Ovid databases for randomized controlled trials evaluating eHealth interventions targeting immunosuppressant medication adherence in kidney transplant recipients. The search time frame spanned from database inception to November 2024. Two investigators independently screened studies, extracted data, and assessed outcomes. Primary outcomes included adherence measured by self-reported questionnaires, electronic monitoring devices, tacrolimus trough levels, intrapatient variability of tacrolimus concentrations, and the proportion of patients achieving a tacrolimus coefficient of variation <40%. Meta-analyses were performed for dichotomous data and continuous data, while narrative synthesis was applied to single studies or data unsuitable for meta-analysis. Subgroup analyses were conducted to determine whether results differed based on adherence assessment methods, follow-up duration, eHealth functionalities, delivery modes, and intervention designs. Risk of bias and evidence quality were evaluated using the Cochrane Risk of Bias 2 tool and the Grading of Recommendations, Assessment, Development, and Evaluation approach, respectively.

**Results:**

A total of 12 studies involving 1234 kidney transplant recipients were included. Significant between-group differences in adherence were observed only when assessed via electronic monitoring devices (risk ratio=1.46, *P*=.006; mean difference=0.37, *P*<.001). However, sensitivity analyses using the leave-one-out method demonstrated instability in these findings. Conflicting results or nonsignificant differences (*P*>.05) were identified across other outcome measures and subgroup analyses.

**Conclusions:**

No definitive conclusions can be drawn regarding the efficacy of eHealth interventions in improving medication adherence among kidney transplant recipients, potentially due to heterogeneity in trial designs, intervention characteristics, user preferences, and variations in adherence definitions and measurement methodologies. These uncertainties are underscored by the low or very low quality of evidence, as assessed using the Grading of Recommendations, Assessment, Development, and Evaluation approach. While eHealth holds promise, methodological refinements in study design and implementation remain critical. Future research should prioritize high-quality, large-scale evidence to validate its clinical efficacy.

**Trial Registration:**

PROSPERO CRD42025640638; https://www.crd.york.ac.uk/PROSPERO/view/CRD42025640638

## Introduction

### Background

According to 2023 statistics from the World Health Organization and the Global Observatory on Donation and Transplantation, approximately 170,000 organ transplants are performed annually worldwide, with kidney transplantation accounting for 64.46% of cases [1]. As the best treatment modality for end-stage renal disease, kidney transplantation demonstrates a 1-year graft survival rate exceeding 95% and significantly enhances patient survival and quality of life compared with dialysis [2]. However, long-term outcomes remain suboptimal, with 10-year graft survival rates declining to 67.8% [3].

Suboptimal medication adherence is recognized as an independent risk factor for these adverse outcomes. Studies report irregular medication-taking behaviors in 15.5% to 57.5% of kidney transplant recipients, with adherence annually declining by 4.8% after transplantation [4,5]. Poor adherence correlates with a 7-fold increased risk of graft failure, a 3-fold higher risk of mortality and exacerbated health care costs [6]. Conventional adherence interventions, such as face-to-face education, clinic follow-ups, or telephone reminders, remain constrained by limited spatiotemporal accessibility, workforce burdens, and patient passivity.

In recent years, eHealth technologies, including SMS text messaging, mobile apps, wearable devices, and artificial intelligence–assisted systems, have emerged as innovative solutions to addressing medication nonadherence [7]. Their core strengths lie in real-time capabilities, personalization, and interactivity. For instance, intelligent reminder systems reduce unintentional nonadherence in patients with cardiovascular diseases during asymptomatic phases [8], remote monitoring facilitates continuous glucose tracking and medication persistence in diabetes management [9], and anonymized virtual counseling mitigates treatment discontinuation caused by stigma among individuals with mental health conditions [10]. Furthermore, algorithm-driven dynamic feedback mechanisms enable precise detection of medication deviations and deliver tailored guidance [11,12], a feature of particular value to kidney transplant recipients requiring long-term complex immunosuppressive regimens [13,14]. Therefore, investigating the efficacy of eHealth technologies in optimizing medication adherence among kidney transplant recipients holds critical research significance for improving long-term graft survival.

Previous studies have extensively explored eHealth-based interventions to improve medication adherence in chronic conditions, such as cardiovascular diseases and stroke [15-17], with meta-analyses demonstrating significant efficacy [18,19]. However, primary research remains limited among kidney transplant recipients, with inconsistent findings across studies [11,12]. A systematic review [7] indicated that short-term eHealth interventions could enhance medication adherence in solid organ transplant recipients, while a separate meta-analysis [20] found eHealth strategies comparable to standard care in improving adherence. Notably, these meta-analyses did not specifically focus on kidney transplant recipients but included heterogeneous transplant populations (liver, kidney, lung, and heart), thus offering limited generalizability to kidney transplantation. Furthermore, few studies have evaluated long-term biochemical outcomes, such as tacrolimus trough levels or intrapatient variability (IPV) of tacrolimus concentrations.

### Objectives

This study conducted a systematic review and meta-analysis focusing on kidney transplant recipients, incorporating both subjective and objective behavioral data alongside biomarkers (eg, tacrolimus IPV). The objectives were to comprehensively evaluate the effectiveness of eHealth interventions in enhancing medication adherence within this population and to explore potential confounding factors, thereby providing an evidence-based foundation for developing precision-driven, sustainable eHealth-based medication adherence management strategies in clinical practice.

## Methods

### Design

This study has been registered with PROSPERO (CRD42025640638; January 31, 2025). The review was conducted in accordance with PRISMA (Preferred Reporting Items for Systematic Reviews and Meta-Analyses) guidelines (Multimedia Appendix 1).

### Ethical Considerations

Ethics approval was not required for this systematic review by the authors’ institutional review board, as it exclusively analyzed existing published data.

### Search Strategy

Before data collection, we formulated the research questions using the Population, Intervention, Comparator, Outcome, and Study Design framework. Subsequently, keywords were extracted from these research questions, with English terms derived from Medical Subject Headings. Search concepts included “kidney transplantation,” “medication adherence,” “telemedicine,” “smartphone,” “mobile applications,” “Internet,” “self-management,” and “randomized controlled trials.” A comprehensive search strategy incorporating these keywords and conceptual synonyms was implemented across 7 electronic databases (PubMed, Embase, Web of Science, CINAHL, Scopus, Ovid, and Cochrane Library), covering records from database inception through November 19, 2024 (Table S1 in Multimedia Appendix 2). In addition, manual reference screening of included articles was performed to identify additional relevant sources.

### Study Selection

Inclusion criteria were established based on the population, intervention, comparator, outcome, and study design framework. Studies meeting the criteria provided in Textbox 1 were included.

**Inclusion criteria based on the population, intervention, comparator, outcome, and study design framework.**

- Participants: kidney transplant recipients receiving immunosuppressive therapy
- Interventions: the experimental group received eHealth interventions, defined as interventions delivered via information and communication technologies (eg, telephone calls; SMS text messaging; telemedicine, including video consultations; websites; and mobile apps)
- Comparison: the control group received any form of conventional care without eHealth components targeting medication adherence improvement
- Outcomes: medication adherence levels in kidney transplant recipients were assessed through objective and subjective measures. Objective measures included electronically monitored adherence, tacrolimus trough levels, intrapatient variability of tacrolimus concentrations (expressed as coefficient of variation = [SD/mean] × 100%), and the proportion of patients achieving tacrolimus coefficient of variation<40% (validated as the threshold distinguishing normal from elevated intrapatient variability in previous studies). Subjective measures included self-reported adherence via validated scales (eg, Basel Assessment of Adherence to Immunosuppressive Medication Scale, Immunosuppressive Medication Adherence Scale, and Medical Adherence Measure Medication Module).
- Study design: randomized controlled trials exclusively
- Outcome definitions: medication adherence (adherence rates or scores directly reported in original studies); taking adherence (proportion of prescribed doses consumed); timing adherence (proportion of doses taken within prescribed dosing intervals)

Given the current lack of reliable access to unpublished study data and concerns about potential biases in nonpeer-reviewed research [21,22], this review exclusively included peer-reviewed articles. In addition, studies were excluded if they were trial protocols, non-English publications, abstract-only reports, or studies without accessible full text.

Following a review of intervention components, we categorized eHealth functionalities into the following domains:

- Educational, defined as interventions that aimed to provide systematic instruction and knowledge dissemination about medication use, including instructional videos, cell phone apps, and educational SMS text messages
- Reminders, that is, memory-enhancing interventions, including electronic pillboxes, cell phone texting, phones, apps, and sensors
- Self-monitoring and feedback, comprising interventions that aimed to analyze existing behaviors and compare them to goals, resulting in retrospective reports (the foundation for behavior change), include telehealth, mobile apps, and the Internet of Things
- Behavioral counseling, composed of interventions that aimed to systematically communicate and assess medication behaviors to help patients identify and analyze medication problems, including telemedicine video, telephone counseling, and websites
- Multifunctional, in other words, interventions integrating ≥2 of the above eHealth functionalities

### Data Extraction

All retrieved records were managed using the EndNote (Clarivate) software, with duplicate entries systematically removed. Two investigators (LZ and LC) independently screened titles and abstracts, followed by a full-text review of potentially eligible studies for final inclusion. Discrepancies were resolved through consensus discussions, with arbitration by a third independent assessor (XH) when required. Data extraction encompassed first author, publication year, country, sample size, mean participant age, intervention components, follow-up duration, measurement time points, and outcome metrics.

### Quality Assessment

Two reviewers (LZ and LC) independently assessed the methodological quality of the included randomized controlled trials (RCTs) using the Cochrane Risk of Bias (ROB) 2 tool [23]. This tool evaluates five domains: (1) randomization process, (2) deviations from intended interventions, (3) missing outcome data, (4) outcome measurement, and (5) selective outcome reporting. For each domain, judgments of “low risk of bias,” “some concerns,” or “high risk of bias” were assigned based on responses to signaling questions. The overall study-level risk of bias was determined by synthesizing domain-specific evaluations in accordance with ROB 2 guidelines. Discrepancies were resolved through discussion with a third reviewer (XH).

The quality of evidence was appraised using the Grading of Recommendations, Assessment, Development, and Evaluation (GRADE) framework, categorizing evidence into 4 levels: high, moderate, low, or very low [24]. RCTs initially received a “high” rating, which was subsequently downgraded based on the risk of bias, imprecision (eg, small sample size), inconsistency (eg, *I*^2^>50%), indirectness, or publication bias [25-29].

### Statistical Analysis

Meta-analyses were conducted using RevMan software (version 5.4; Cochrane). Continuous outcomes were analyzed via standardized mean difference (SMD) or weighted mean difference (MD), while dichotomous outcomes were evaluated using risk ratio (RR), all reported with 95% CI. A 2-tailed *P*<.05 was considered statistically significant. Heterogeneity was assessed using the Cochran Q test (significance threshold *P*<.10) and quantified via the *I*^2^ statistic [30]. A random-effects model was applied when *I*^2^>50% and *P*<.10; otherwise, a fixed-effects model was used. Sensitivity analyses (leave-one-out method) evaluated the robustness of the primary findings [31]. Publication bias was examined via funnel plots with the Egger test when ≥10 studies were included [32]. Subgroup analyses addressed clinical and methodological heterogeneity based on the following measures:

1. Adherence assessment method (electronic monitoring, self-report, and others)
2. Follow-up duration (≤3 months, >3-<6 months, and >6-12 months)
3. eHealth functionality (multifunctional vs single function)
4. Delivery mode (mixed mode vs single mode)
5. Intervention design (theory based vs not theory based [or unreported]; patient involved vs not involved [or unreported])

Meta-analyses were performed for dichotomous and continuous data, whereas narrative synthesis was applied to single studies or data unsuitable for pooling.

## Results

### Study Selection

**Figure 1:**
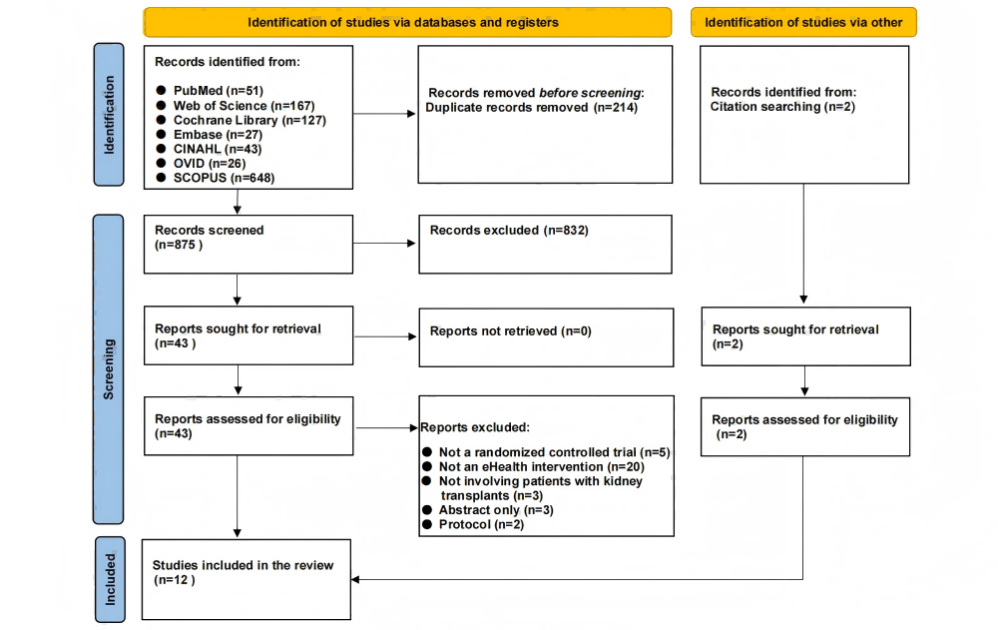
PRISMA flow diagram.

The initial search produced 1089 records, of which 214 (19.65%) were removed due to duplication. On the basis of the assessment of article titles and abstracts, an additional 832 (76.4%) irrelevant records were removed, and 43 (3.95%) article papers were ultimately selected for the full review. Of these 43 papers, 10 (23.3%) were selected for the systematic review. By integrating 2 additional papers obtained from different references, 12 papers were included in this study. The study selection process is summarized in the PRISMA-compliant flow diagram (Figure 1).

### Study Characteristics

The characteristics of the included studies are provided in Table 1. A total of 12 studies involving 1234 patients were included in this systematic evaluation to assess the effectiveness of eHealth interventions on medication adherence in renal transplant recipients. All studies were RCTs published between 2013 and 2025. Of the 12 studies, 4 (33%) were conducted in the United States, 1 (8%) in Turkey, 1 (8%) in Australia, 2 (17%) in South Korea, 1 (8%) in Germany, 1 (8%) in Sweden, and 2 (17%) in Canada. The duration of follow-up ranged from 3 to 12 months.

**Table 1:** Descriptive summary of included studies.

Study, year; country	Sample size, n		Age (y), mean (SD) or median (IQR)		Intervention (duration)	Control	Measurement point (mo)	Outcome measures (medication adherence)
	Intervention	Control	Intervention	Control				
Erdal and Karazeybek [33], 2025; Turkey	50	50	40.8 (11.67)	42.9 (13.67)	SMS medication reminders and medication education (3 mo)	No intervention	1, 2, and 3	Objective: tacrolimus blood concentration Subjective: IMAS^a^ (self-report questionnaire)
McGillicuddy et al [34], 2013; United States	9	10	42.4 (12.04)	57.6 (8.28)	Electronic pill tray reminder; SMS text message, email, or phone call (noncompliance); and electronic feedback from the physician (3 mo)	Routine posttransplant care and education	1, 2, and 3	Objective: record electronic pill tray openings using the adherence score proposed by Russell et al [35]
Fleming et al [12], 2021; United States	68	68	50.2 (12.3)	51.2 (13.7)	TRANSAFE Rx (Improving Transplant Medication Safety Through a Pharmacist-Empowered) app reminder and pharmacist feedback (12 mo)	Routine care	Monthly measurements for 12 mo	Objective: percentage of patients with tacrolimus CV^b^<40%
McGillicuddy et al [36], 2020; United States	41	41	52.1 (11.3)	51.5 (12.5)	Electronic pill tray reminder; SMS text message, email, or phone call (noncompliance); and electronic feedback (12 mo)	Routine posttransplant care and education and electronic drug trays without reminder	1, 3, and 6	Objective: tacrolimus IPV^c^, percentage of patients with tacrolimus CV<40%, and electronic monitoring (electronic drug tray compartment openings)
Low et al [37], 2019; Australia	35	36	53.6 (11.3)	48.4 (11.1)	Medication assessment and consumer-centered video, health coaching, and MEMS^d^ (12 mo)	Standard care and MEMS	3, 6, 9, and 12	Objective: electronic monitoring (MEMS monitoring of vial openings) Subjective: BAASIS^e^ (self-report questionnaire)
Han et al [38], 2019; South Korea	70	66	45.0 (35-54)	43.0 (30-52)	Adhere4U app, which provides medication reminders, tracking, reporting, education, and laboratory results, and MEMS (6 mo)	Routine care and MEMS	1, 3, and 6	Objective: electronic monitoring (MEMS monitoring of vial openings) Subjective: BAASIS and VAS^f^ (self-report questionnaire)
Schmid et al [39], 2017; Germany	23	23	46.0 (18-59)	51.0 (19-66)	Remote monitoring and real-time video consultation by the medical team (12 mo)	Routine care and education	3, 6, and 12	CAS^g^ for tacrolimus blood concentration, clinician’s report score, and BAASIS (self-report questionnaire)
Henriksson et al [40], 2016; Sweden	40	40	44.3 (9-68)	45.0 (2-69)	EMD^h^ reminder to take medication and report generation (12 mo)	Standard care	10 visits in 1 year (baseline; day 7-14; and week 4, 8, 12, 16, 20, 24, 36, and 52)	Objective: tacrolimus blood concentration and electronic monitoring (EMD monitoring of vial openings)
Mansell et al [41], 2024; Canada	91	82	52.6 (11.2)	49.4 (13.3)	eVideo education and signed medication contract (12 mo)	Routine education	3 and 12	Objective: tacrolimus CV Subjective: BAASIS and VAS (self-report questionnaire)
Reese et al [42], 2017; United States	39^i^ and 40^j^	38	50.0^i^ (11.0) and 50.0^j^ (12.0)	49.0 (11.0)	Group 1: reminded to take medication by the physician and electronic pill bottle; group 2: reminded to take medication by electronic pill bottle only (6 mo)	Electronic pill bottles with no reminder function and recording only medication adherence data	6	Objective: tacrolimus CV, tacrolimus blood concentration, and electronic monitoring (electronic pill bottle openings) Subjective: BAASIS (self-report questionnaire)
Jung et al [11], 2020; South Korea	51	54	49.9 (10.0)	49.0 (12.2)	Electronic pillbox reminder, home monitoring, data transfer to EMR^k^, and SMS text message to alert medical staff and patients when medication is missed or wrongly taken (6 mo)	Outpatient follow-up	Week 4, 8, 16, 20, and 24	Objective: tacrolimus CV and tacrolimus blood concentration
Foster et al [43], 2018; Canada	81	88	15.5 (13.3-17.5)	15.8 (13.2-17.4)	Electronic pillbox reminders, tracking of medication behaviors, support group review, and education (12 mo)	3 monthly meetings with a coach who only listens and offers general help	3, 6, 9, and 12	Objective: electronic monitoring (electronic pillbox openings) Subjective: MAM-MM^l^ (self-report questionnaire)

^a^IMAS: Immunosuppressive Medication Adherence Scale.

^b^CV: coefficient of variation.

^c^IPV: intrapatient variability.

^d^MEMS: Medication Event Monitoring System (the electronic microprocessor embedded in the medication bottle cap monitors medication adherence by detecting cap-opening events, although it lacks reminder functionality).

^e^BAASIS: Basel Assessment of Adherence to Immunosuppressive Medication Scale.

^f^VAS: Visual Analog Scale.

^g^CAS: Composite Adherence Score.

^h^EMD: electronic medication dispenser.

^i^Intervention group 1.

^j^Intervention group 2.

^k^EMR: electronic medical record.

^l^MAM-MM: Medical Adherence Measure Medication Module.

Methods used to assess medication adherence were categorized as subjective or objective. Subjective measures were based on self-reported assessments in 6 (50%) studies. Objective measures included electronic monitoring methods in 8 (67%) studies, tacrolimus blood concentrations in 4 (33%) studies, tacrolimus coefficients of variation (CVs) in 5 (42%) studies, and the percentage of patients with tacrolimus CV<40% in 2 (17%) studies. Another study used a comprehensive measure combining subjective and objective measures. eHealth had a variety of functions, intervention designs, and interactions (Tables S1 and S2 in Multimedia Appendix 2): 10 (83%) studies used a mixed-delivery model and 2 (17%) used a single delivery model; 10 (83%) studies had multifunctional eHealth interventions and 2 (17%) had single-function eHealth interventions; 8 (67%) studies involved patients in the intervention design while 4 (33%) did not or were unreported; and 6 (50%) studies had interventions based on behavioral theories while the remaining 6 (50%) had no theoretical basis or were unreported. We also summarized qualitatively other characteristics of each study (eg, user concerns and usability assessment), as detailed in Tables S1 and S2 in Multimedia Appendix 2.

In the study by Erdal and Karazeybek [33], the intervention group received immunosuppressant drug reminders 4 times a day and drug education SMS text messages on Mondays, Wednesdays, and Fridays for 3 months. In the study by McGillicuddy et al [34], on the prescribed dosage day and time, the intervention group received customized reminder signals (lights, chimes, phone calls, or SMS text messages). When medication nonadherence alerts occurred, they received SMS text messages, emails, or calls. A weekly email report summarized participants’ adherence to physician-prescribed drug doses. In the study by Fleming et al [12], intervention group participants received personalized prescription regimens, medication reminders, and monthly summary comments from the self-developed TRANSAFE Rx app. Pharmacists analyzed and changed medication according to summary reports. In the study by McGillicuddy et al [36], the electronic drug tray used lights, bells, phone calls, or SMS text messages to remind the intervention group to take their prescriptions on time and provide medication adherence data from the previous day. Back-office health care providers evaluated adherence biweekly. In the study by Low et al [37], biweekly face-to-face meetings (medication assessments and consumer-centered videos) and telephone health coaching were conducted for patients in the intervention group. In the study by Han et al [38], the Adhere4U app was downloaded on the intervention group patients’ phones for medication reminders, tracking, adherence reports, immunosuppressant information, educational films, and laboratory results. In the study by Schmid et al [39], at discharge, the intervention group started chronic care chart management, and transplant nurses regularly assessed medication intake through online questionnaires, video screen counseling, and telephone calls to provide timely feedback to transplant physicians in response to unexpected emergencies. In the study by Henriksson et al [40], an electronic medication dispenser was used to remind the intervention group patients to take their medication at prescribed times with visual and auditory signals and send regular adherence reports to them. In the study by Mansell et al [41], intervention group participants were required to watch the medication education video, complete self–goal-setting exercise activities on an electronic device, and sign a contract to take medication as prescribed by their physician. In the study by Reese et al [42], the smart pillbox flashed and beeped to remind participants in intervention groups 1 and 2 to take their medication. If intervention group 1 patients had <90% medication adherence (measured every 2 weeks), the study coordinator and nephrologist called them. In the study by Jung et al [11], pillboxes reminded the intervention group to take their medications at the right time, and the home monitoring device sent pillbox medication data to the electronic medical record for integration and analysis. SMS text message alerts notified medical professionals and patients of missed or incorrect doses. In the study by Foster et al [43], a medication adherence support group consisting of patients, coaches (specially trained clinical investigators), and parents convened quarterly to analyze adherence data and identify obstacles to medication use. Patients were permitted to select their preferred medicine reminders via SMS text message, email, or visual cues.

### Definition of Medication Adherence

The definition of *medication adherence* varied from study to study, with adherence defined as follows. Erdal and Karazeybek [33] assessed medication adherence using the Immunosuppressive Medication Adherence Scale self-adherence evaluation (continuous data) and tacrolimus blood concentrations (dichotomized data). The 11-item self-report Immunosuppressive Medication Adherence Scale assessed immunosuppressive medicine adherence. A total of 8 items were rated on a 5-point Likert scale from “never, rarely, sometimes, often, and always.” Yes or no were the responses for the remaining 3 questions. Higher item scores promote medication adherence. Total scores were 11 to 55 and came from item scores, with Cronbach α=0.61. Tacrolimus blood levels in patients were measured 12 hours following medication. The mean tacrolimus plasma concentration was estimated from blood samples at 1, 2, and 3 months after the intervention. This study defined “medication nonadherence” as a tacrolimus level <5 from >10 ng/mL.

McGillicuddy et al [34] objectively measured medication time using the adherence score by Russell et al [35] Medication adherence was taking immunosuppressants within 3 hours of the dose. Doses taken within 3 hours received a full score, those taken within 6 hours received a half score, and missed doses received 0 points. Participants received daily scores from 0.0 to 1.0, which were averaged over a month.

Fleming et al [12] investigated medication adherence using 2 objective physiological indicators: tacrolimus IPV and the percentage of patients with an IPV <40%. Tacrolimus IPV was measured using CV ([mean/SD] × 100) at monthly intervals for each patient (12-month rolling average), beginning with randomization and lasting 12 months. Rolling averages were calculated and estimated at monthly intervals. Each month, 1 months’ worth of tacrolimus levels was added to the CV calculation, while the preceding month’s levels were removed. In this study, a narrative synthesis of the 2 outcomes was conducted. In a 2020 study by McGillicuddy et al [36], the Russell et al [35] adherence score (dichotomized data) was used to objectively assess patient adherence to medication using tacrolimus IPV, the percentage of patients with tacrolimus IPV <40%, and medication administration data monitored by an electronic medication tray.

The study by Han et al [38] investigated medication adherence via the Basel Assessment of Adherence to Immunosuppressive Medication Scale (BAASIS) and Visual Analog Scale (VAS; dichotomized data), as well as the objective method of Medication Event Monitoring System (MEMS) prescription container lids (dichotomized data). A total of 4 items on the BAASIS examined medication dosage, duration, holidays, and tapering on a 6-point scale from “never” (0 points) to “every day” (5 points). VAS values ranged from 0 (never take medication as prescribed) to 100 (always take medication as prescribed). The BAASIS classified noncompliance as a score ≥1 on any of the 4 categories, while the VAS defined it as a score <100. Our study used values measured by BAASIS. The objective findings were binary indicators of 6-month cumulative adherence based on electronic monitoring (MEMS) data. Medication nonadherence was defined as adherence <98%, >102%, or at least 1 medication holiday. The top limit of 102% relates to repeated overdosing, while medication holidays are durations without drug ingestion >3 times the normal interval. Furthermore, Low et al [37] used the BAASIS and MEMS prescription container lids to assess medication adherence. Patients with ≥97% “taking adherence” and “timing adherence” were considered adherent. Taking adherence is the proportion of days with the correct number of MEMS openings divided by the total monitored days. Timing adherence is the percentage of days the MEMS was opened within 2 hours of the patient’s average medication-taking time.

In the study by Schmid et al [39], nonadherence was measured with a Composite Adherence Score (CAS), which included a self-report, 2 clinician reports, and a tacrolimus test report. The nonadherence was determined using the CAS threshold system defined by Schäfer-Keller et al [44]. To better understand the dynamics of nonadherence, they changed the CAS percentage grading into an interval grading system and a description of nonadherence, which were assessed at baseline and 3, 6, and 12 months after the intervention.

Henriksson et al [40] used objective markers to assess medication adherence, such as using an electronic dispenser to track whether patients took their prescribed medication on time and measure tacrolimus levels. Mansell et al [41] assessed medication adherence using subjective and objective measures, such as the BAASIS (dichotomized data), VAS, and tacrolimus IPV. This review used BAASIS and tacrolimus IPV results. Reese et al [42] used 2 types of measurements to investigate medication adherence: objective data from electronic pill bottles, tacrolimus blood concentration, and tacrolimus IPV (continuous data) and subjective data from the BAASIS for self-rated adherence (dichotomized data). Medication adherence was determined by the number of days when the bottle was opened at the appropriate time for daily tacrolimus dosages, tacrolimus blood concentrations, and BAASIS scores using a validated 5-item self-report questionnaire intended for immunosuppression.

Jung et al [11] conducted their study using a variety of objective metrics. For example, tacrolimus blood levels (continuous data), tacrolimus IPV (continuous data), and electronic pillboxes were used to record medication adherence data, such as dose-taking adherence, dose-frequency adherence, dose-interval adherence, and drug holidays. Dose adherence was computed as follows: (number of pills ingested during a specified timeframe/number of pills prescribed within the same timeframe) × 100%. Dose-frequency adherence was determined by the formula: (number of days with accurate daily dosage in a specified time frame/number of days within the same time frame). Dosing interval adherence was determined by the formula: (number of days with accurate dosing intervals within a certain time frame/number of days in the same time frame) × 100%. A range of –25% to +25% was used to delineate the appropriate dosing interval. In their research, the medication was administered bidaily with a 12-hour dose interval. The acceptable dose-interval range was 9 to 15 hours. Drug holidays were calculated as follows: (days not taken/number of days in the same time) × 100%.

In the study by Foster et al [43], medication adherence was categorized into “taking” adherence and “timing” adherence. The main result was a record of electronic equipment. Secondary outcomes included the Medical Adherence Measure Medication Module (MAM-MM) self-reported adherence (continuous data) [45]. The MAM-MM measured self-reported taking and timing adherence by counting doses taken in the previous week and 2 hours after the suggested time. Each patient’s MAM-MM score was averaged for 4 postintervention months (3, 6, 9, and 12). This review used the MAM-MM self-assessment and e-measurement outcomes.

### Risk of Bias

To assess the risk of bias in each study, we used the revised Cochrane ROB 2 tool for RCTs. A summary of the risk of bias assessments for included studies is presented in Figures S1 and S2 in Multimedia Appendix 3. Of the 12 studies, 11 (92%) [11,12,33,34,36-38,40-43] were rated as having “some concerns,” while 1 (8%) study [39] was classified as “high risk.” A total of 6 (50%) studies [11,12,34,36,42,43] failed to provide adequate allocation concealment details, with 4 (33%) [12,34,36,42] merely stating “randomized allocation” without methodological specifics. Blinding of participants and personnel was inherently challenging due to the pragmatic nature and clinical context of the interventions, resulting in “some concerns” in domain 2 (deviations from intended interventions). No studies exhibited missing outcome data. One study [39] lacked assessor blinding and used a CAS integrating patient self-reports, dual clinician evaluations, and tacrolimus level assessments. Clinicians being unblinded to group allocation led to a “high risk” rating in domain 4 (measurement of the outcome). Outcome measures in the remaining studies relied on patient self-reported data or objective metrics (electronic monitoring and biomarker levels), minimizing assessor influence. No evidence of selective outcome reporting bias was identified.

### Outcomes

#### Medication Adherence Assessed by Self-Report

**Figure 2:**
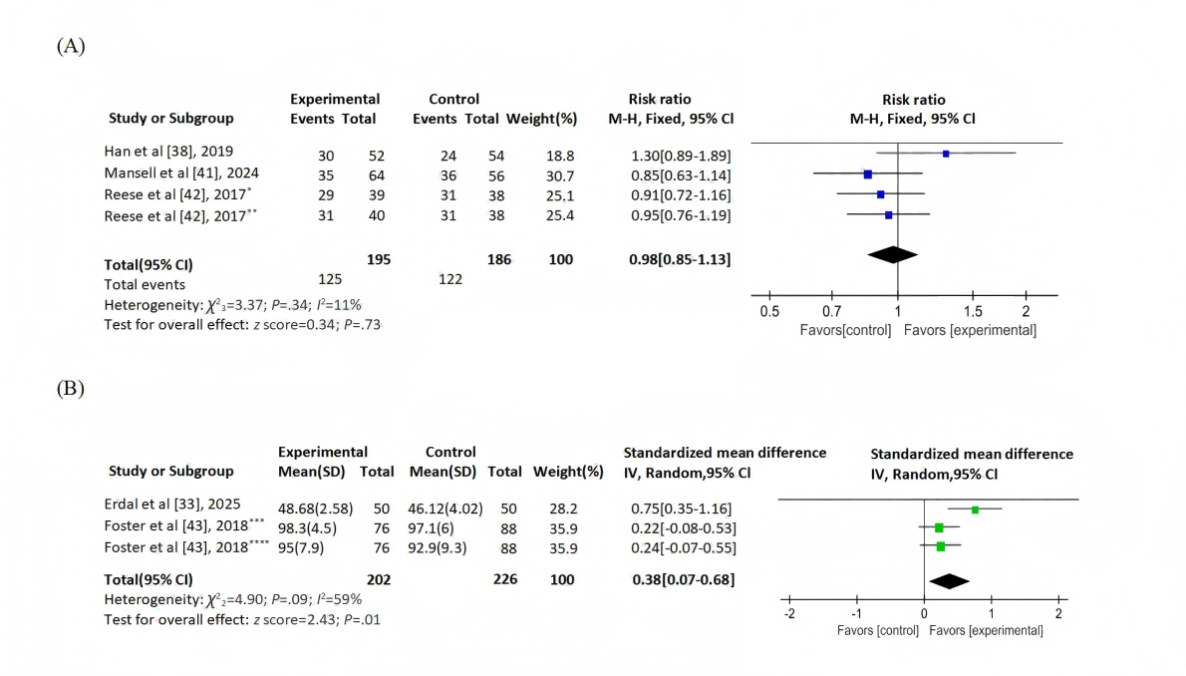
Medication adherence assessed by self-report: (A) dichotomized data and (B) continuous data.*Group 1: medication reminders from physicians and wireless pill bottles; **group 2: medication reminders from wireless pill bottles, ***taking adherence, and ****timing adherence. M-H: Mantel-Haenszel; IV: inverse variance.

Of the 12 studies, 6 (50%) [33,37,38,41-43] used self-reported assessment questionnaires. A total of 4 (33%) studies [37,38,41,42] reported medication adherence measured by BAASIS, with 3 providing analyzable data. Due to low heterogeneity (*I*^2^=11%; *P*=.34), a fixed-effect model was applied. No significant between-group difference was observed (RR=0.98, 95% CI 0.85-1.13; *z* score=0.34; *P*=.73; Figure 2A) [38,41,42]. In total, 17% (2/12) of the studies [33,43] reported continuous adherence scores from distinct self-report tools (*I*^2^=59%; *P*=.09), warranting an SMD random-effects model. The eHealth intervention group showed statistically significant improvement in adherence (SMD 0.38, 95% CI 0.07-0.68; *z* score=2.43; *P*=.01; Figure 2B) [33,43]. One study [37] reported significant intergroup differences (*P*<.001) without providing quantitative data and was thus excluded from the meta-analysis (Table S3 in Multimedia Appendix 2).

#### Medication Adherence Monitored by Electronic Devices

**Figure 3:**
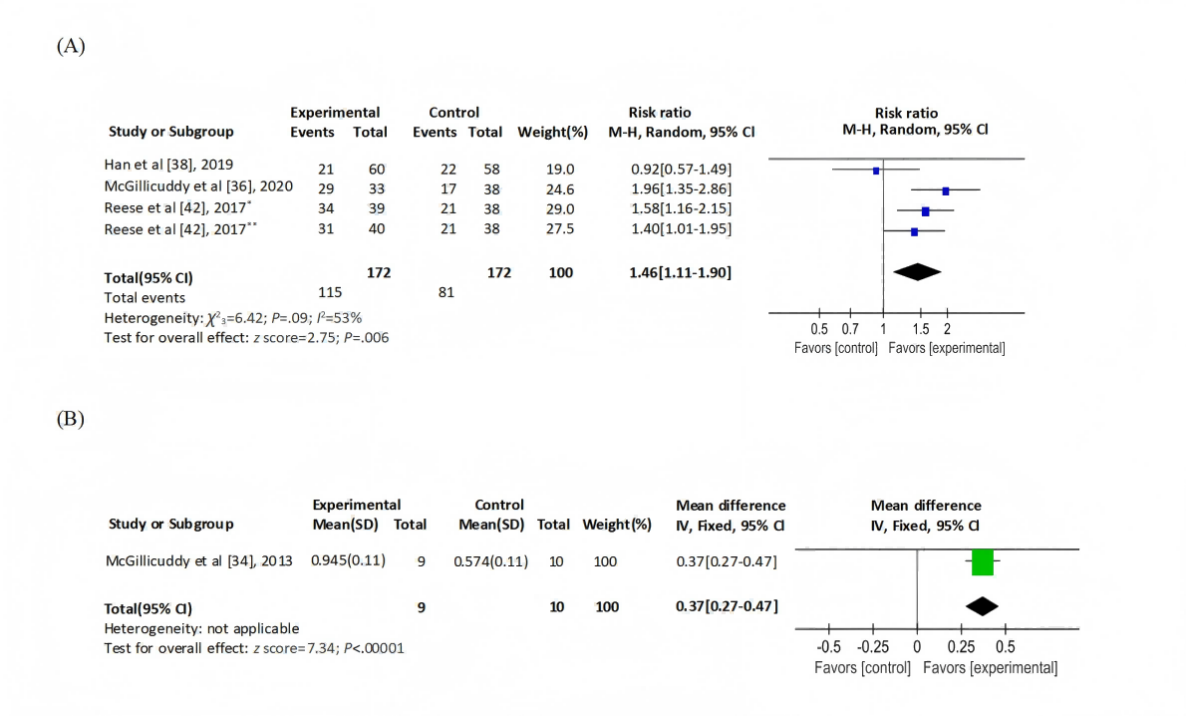
Medication adherence monitored by electronic devices: (A) dichotomized data and (B) continuous data. *Group 1: medication reminders from physicians and wireless pill bottles, **group 2: medication reminders from wireless pill bottles. M-H: Mantel-Haenszel; IV: inverse variance.

Of the 12 studies, 8 (67%) [11,34,36-38,40,42,43] reported medication adherence measured via electronic monitoring devices, with 3 (25%) [36,38,42] providing dichotomous data (*I*^2^=53%; *P*=.09), for which a random-effects model was applied. The eHealth intervention demonstrated statistically significant improvements in adherence (RR=1.46, 95% CI 1.11-1.90; *z* score=2.75; *P*=.01; Figure 3A) [36,38,42]. One study [34] reporting continuous data showed significant between-group differences (MD 0.37, 95% CI 0.27-0.47; *z* score=7.34; *P*<.001; Figure 3B). A total of 4 (33%) studies [11,37,40,43] unsuitable for meta-analysis underwent narrative synthesis (Table S3 in Multimedia Appendix 2): 2 [11,37] reported no substantial intergroup differences, 1 [43] identified significant improvements, and another [40] exclusively reported 97.8% (23,296/23,820) adherence in the intervention group without monitoring the control arm (electronic medication dispensers were not provided to track dosing).

#### Tacrolimus Blood Levels

**Figure 4:**
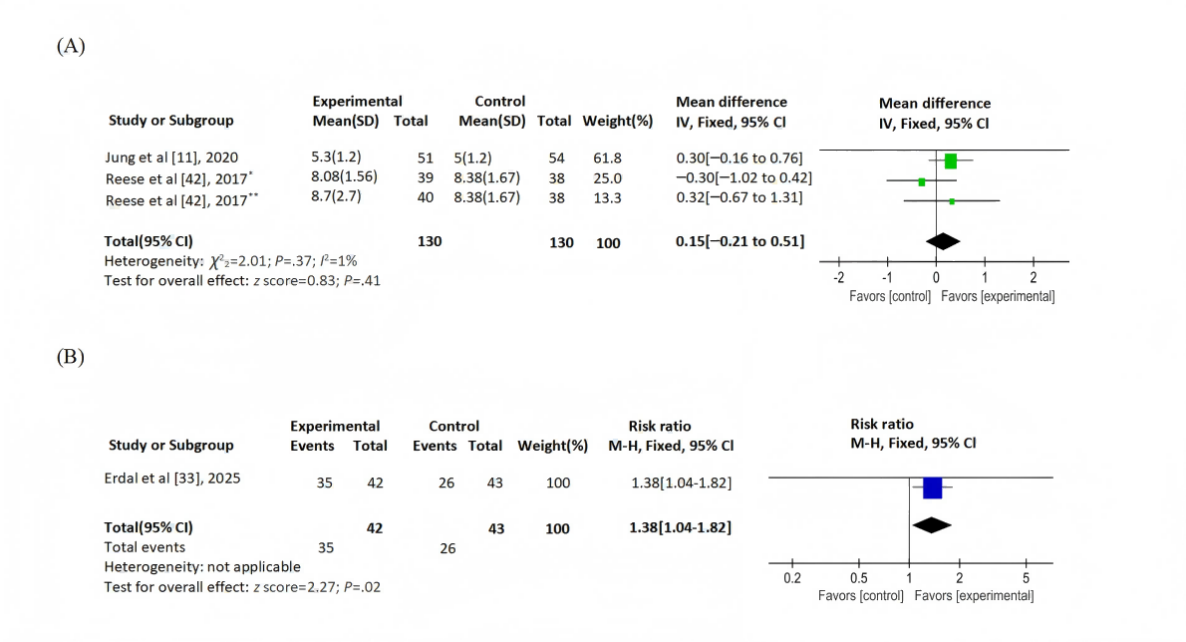
Tacrolimus blood concentration: (A) continuous data and (B) dichotomized data. *Group 1: medication reminders from physicians and wireless pill bottles, **group 2: medication reminders from wireless pill bottles. M-H: Mantel-Haenszel; IV: inverse variance.

Of the 12 studies, 4 (33%) [11,33,40,42] reported tacrolimus trough levels as adherence biomarkers. A total of 2 (17%) studies [11,42] provided continuous outcome data, with minimal heterogeneity (*I*^2^=1%; *P*=.37), warranting a fixed-effect model. No significant between-group differences were observed (MD 0.15, 95% CI –0.21 to 0.51; *z* score=0.83; *P*=.41; Figure 4A) [11,42]. One study [33] reporting dichotomous outcomes demonstrated statistically significant improvements in the intervention group (RR=1.38, 95% CI 1.04-1.82; *z* score=2.27; *P*=.02; Figure 4B). Another study [40] did not provide analyzable data but stated that the difference between the 2 groups was not statistically significant.

#### Tacrolimus IPV

**Figure 5:**
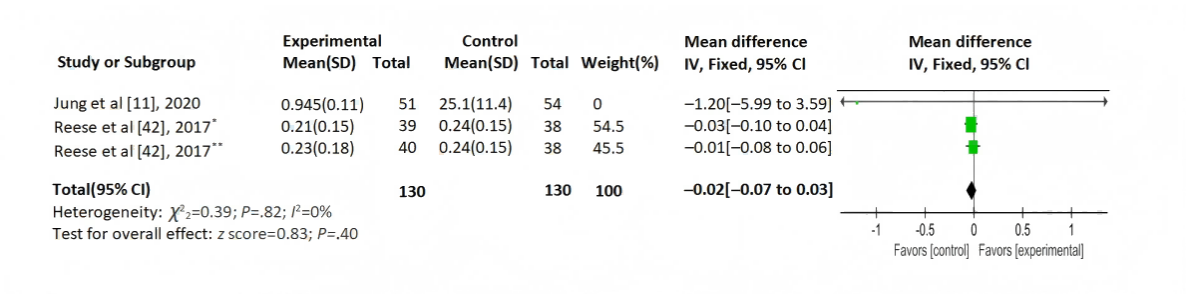
Intrapatient variability of tacrolimus concentrations. *Group 1: medication reminders from physicians and wireless pill bottles, **group 2: medication reminders from wireless pill bottles. IV: inverse variance.

Of the 12 studies, 5 (42%) [11,12,36,41,42] reported the IPV of tacrolimus concentrations. A total of 2 (17%) studies [11,42] provided continuous outcome data with negligible heterogeneity (*I*^2^=0%; *P*=.82), justifying a fixed-effect model. No significant between-group differences were observed (MD –0.02, 95% CI –0.07 to 0.03; *z* score=0.83; *P*=.40; Figure 5). A total of 3 (25%) studies [12,36,41] unsuitable for meta-analysis underwent narrative synthesis (Table S3 Multimedia Appendix 2): 2 [12,36] demonstrated significant between-group differences, while 1 [41] reported nonsignificant results.

#### Proportion of Patients With Tacrolimus CV <40%

This review used the narrative synthesis of 2 studies [12,36]. Fleming et al [12] conducted a 12-month RCT of 136 kidney transplant recipients and found that the control group had a higher proportion of recipients with a tacrolimus CV <40% at baseline (*P*=.02), but the difference between the 2 groups at 12 months was not statistically significant (*P*=.22). However, the value of the tacrolimus CV <30% after 12 months was substantially different between the 2 groups, with the intervention group overrepresenting in reaching this indicator (*P*=.03), and the baseline of the 2 groups was comparable (*P*=.77). McGillicuddy et al [36] showed that more recipients in the intervention group obtained a tacrolimus CV<40% following a 6-month RCT (80% vs 70%; *P*=.001).

#### Comprehensive Scoring of Medication Adherence

**Figure 6:**
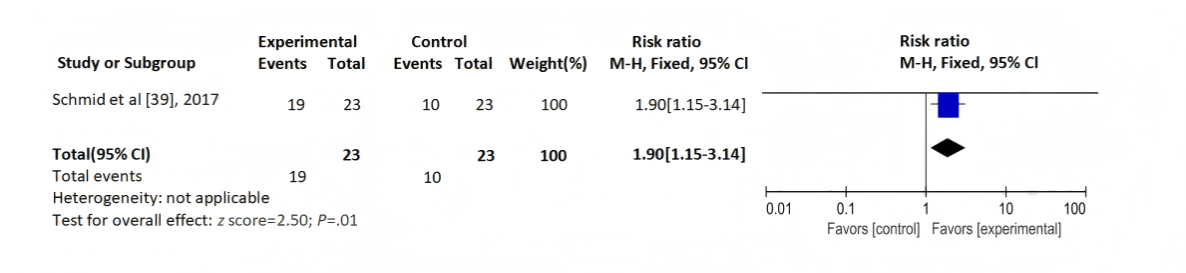
Comprehensive scoring of medication adherence. M-H: Mantel-Haenszel.

In the study by Schmid et al [39], nonadherence was measured by CAS, which included a self-report, 2 clinician reports, and a tacrolimus test report. Their 12-month RCT of 46 patients with kidney transplants found that the intervention group had a lower rate of medication nonadherence compared to the control group (odds ratio=1.90, 95% CI 1.15-3.14; *z* score=2.50; *P*=.01; Figure 6).

### Subgroup Analysis

Owing to insufficient analyzable data and heterogeneity in outcome metrics across studies, pooling all studies was unfeasible. Subgroup analyses were categorized by adherence assessment methods, follow-up duration, eHealth delivery modes, eHealth functionalities, theory-based interventions, and patient involvement in intervention designs. For adherence assessment methods, electronic monitoring demonstrated statistically significant improvements in both dichotomous and continuous outcomes (RR=1.46, 95% CI 1.11-1.90; *z* score=2.75; *P*=.006 and SMD 3.22, 95% CI 1.76-4.68; *z* score=4.32; *P*<.001; Figure S3 in Multimedia Appendix 3 and Table 2). Other subgroup analyses yielded discordant outcomes (significant in 1 metric but nonsignificant in another) or uniformly nonsignificant results across both dichotomous and continuous data (Table 2; Figures S4-S8 in Multimedia Appendix 3). Further analysis of intervention components was precluded by the absence of process evaluations to identify active components driving efficacy in multifunctional or mixed-delivery interventions [46].

**Table 2:** Results of subgroup analyses (N=12 studies).

Outcome metrics and categories		Included studies, n (%)		Sample size in meta-analysis, n	Heterogeneity		Effect size			
					*I*^2^ (%)	*P* value	RR^a^ (95% CI)	SMD^b^ (95% CI)	*z* score	*P* value
**Medication adherence assessment indicators**										
	Self-report	3 (25)	343		11	.34	0.96 (0.83-1.10)	—^c^	0.62	.53
	Self-report	2 (17)	264		59	.09	—	0.38 (0.07-0.68)	2.43	.01^d^
	Electronic monitoring	3 (25)	306		53	.09	1.46 (1.11-1.90)	—	2.75	.006^d^
	Electronic monitoring	1 (8)	19		—	—	—	3.22 (1.76-4.68)	4.32	<.001^d^
	Comprehensive scoring	1 (8)	46		—	—	1.90 (1.15-3.14)	—	2.50	.01^d^
	Others	2 (17)	131		20	.26	1.23 (1.13-2.02)	—	2.79	.005^d^
	Others	4 (33)	222		0	.60	—	–0.01 (0.19-0.16)	0.16	.87
**Follow-up time (mo)**										
	≤3	1 (8)	85		—	—	1.38 (1.04-1.82)		2.27	.02^d^
	≤3	2 (17)	119		90	.001	—	1.88 (0.53-4.29)	1.53	.13
	>3 to ≤6	3 (25)	325		73	.001	1.23 (0.98-1.55)	—	1.79	.07
	>3 to ≤6	2 (17)	222		0	.60	—	–0.01 (0.19-0.16)	0.16	.87
	>6 to ≤12	2 (17)	166		85	.009	1.25 (0.59-2.68)	—	0.59	.56
	>6 to ≤12	1 (8)	164		0	.94	—	0.23 (0.01-0.45)	2.09	.04^d^
**eHealth function**										
	Multifunctional	5 (42)	443		72	.0003	1.22 (0.98-1.51)	—	1.76	.08
	Multifunctional	5 (42)	505		73	.0001	0.20 (0.05-0.46)	0.20 (0.05-0.46)	1.57	.12
	Single function	1 (8)	120		—	—	0.85 (0.63-1.14)	—	1.07	.28
**eHealth delivery mode**										
	Mixed	4 (33)	352		72	.0007	1.28 (1.03-1.60)	—	2.18	.03^d^
	Mixed	4 (33)	405		67	.002	—	0.13 (0.12-0.37)	1.02	.31
	Single	2 (17)	211		0	.55	1.17 (0.95-1.43)	—	1.64	.10
	Single	1 (8)	100		—	—	—	0.75 (0.35-1.16)	3.63	<.001^d^
**Theory-based intervention**										
	Theory based	3 (25)	309		79	.0002	1.19 (0.92-1.54)	—	1.33	.18
	Theory based	3 (25)	300		74	.0009	—	0.17 (0.16-0.49)	1.00	.32
	Not theory based or not reported	3 (25)	255		66	.03	1.13 (0.76-1.67)	—	0.61	.54
	Not theory based or not reported	2 (17)	205		78	.01	—	0.30 (0.18-0.77)	1.22	.22
**User-centered design**										
	Patient involvement	3 (25)	234		80	.0002	1.34 (1.01-1.78)	—	2.05	.04^d^
	Patient involvement	4 (33)	405		67	.002	—	0.13 (0.12-0.37)	1.02	.31
	No patient involvement or not reported	3 (25)	317		56	.11	0.94 (0.68-1.30)	—	0.40	.69
	No patient involvement or not reported	1 (8)	100		—	—	—	0.75 (0.35-1.16)	3.63	<.001^d^

^a^RR: risk ratio.

^b^SMD: standardized mean difference.

^c^Not applicable.

^d^The difference between the two groups was statistically significant (*P*<.05).

### Sensitivity Analysis

Sensitivity analyses (leave-one-out method) were performed for dichotomous and continuous outcomes of self-reported adherence, electronic monitoring, tacrolimus trough levels, tacrolimus IPV, and the proportion of patients achieving tacrolimus CV<40%. The results of the sensitivity analyses are provided in Table S4 in Multimedia Appendix 2. The adherence outcomes derived from electronic monitoring (dichotomous data) and self-reports (continuous data) were not robust, as the exclusion of individual studies led to significant alterations in effect estimates. Pooled results for other metrics remained stable across all iterations, demonstrating robustness.

### Quality of Evidence

The quality of evidence ranged from low to very low due to risk of bias, imprecision, and inconsistency in the included trials (Table S5 in Multimedia Appendix 2). None of the studies were downgraded for indirectness and publication bias.

## Discussion

### Principal Findings

This systematic review and meta-analysis of 12 RCTs demonstrates limited evidence supporting the efficacy of eHealth interventions in improving medication adherence among kidney transplant recipients. A key finding was that the difference in adherence between intervention and control groups was statistically significant (*P*<.05) when tested using electronic monitoring. However, sensitivity analysis revealed that this finding lacked robustness (Table S4 in Multimedia Appendix 2). Other outcome measures and subgroup analyses yielded either conflicting results or uniformly nonsignificant differences (Table S3 in Multimedia Appendix 2 and Table 2). All conclusions were based on “low” to “very low” GRADE quality scores (Table S5 in Multimedia Appendix 2), with most outcomes supported by small sample sizes or single-study data.

This study diverges from previous meta-analyses demonstrating eHealth efficacy in chronic disease management (eg, hypertension: effect size of 0.41, *P*=.04 [47]; diabetes: effect size of 0.501, *P*<.001 [48]), despite similar long-term medication management profiles between kidney transplant recipients and these populations. The mixed results of this review may be attributed to the diverse measures, intervention characteristics, trial designs, and user preferences, as well as to individual trials that led to nonsignificant analyses of effects. Consequently, it is important to focus on the analysis of confounding factors when interpreting the results and to be alert to the risk of false negatives due to methodological limitations. Specific influencing factors are discussed subsequently.

Medication adherence outcomes are susceptible to measurement variability across assessment methodologies. Each adherence assessment modality has inherent strengths and limitations. While multiple methods, including validated questionnaires, blood drug level assays, electronic monitoring, and pill counts, demonstrate clinical utility [49,50], 2 (17%) of the 12 included studies suggest that self-reporting may overestimate actual adherence levels [34,43]. Both intentional and unintentional nonadherence can only be captured through self-reported measures, which are intrinsically vulnerable to social desirability bias (eg, patients may underreport nonadherence to avoid clinician criticism) and recall bias [43,51-53]. In addition, while tacrolimus levels and IPV serve as nonadherence biomarkers in numerous studies [54,55], their interpretation is confounded by external factors and inherent pharmacokinetic variability [33,56]. Despite being regarded as the gold standard for adherence measurement due to its high sensitivity [57], electronic monitoring exhibits notable technical limitations. A total of 2 (17%) of the 12 included studies reported instances where electronic medication dispensers failed to confirm actual drug ingestion (eg, device openings may reflect refill behaviors rather than dose consumption) [11,42], consistent with previous literature [58,59]. Notably, while our analysis identified significant between-group adherence differences via electronic monitoring (Table 2), sensitivity analyses demonstrated instability in these findings (Table S4 in Multimedia Appendix 2). This may stem not only from inherent device limitations but also from insufficient sample sizes (only 3 studies) and intervention heterogeneity. Consequently, we recommend adopting multidimensional assessment strategies (eg, integrating self-reports, electronic monitoring, and tacrolimus trough levels [60]), although feasibility must consider the increased costs and resource demands associated with electronic devices [61]. Future research should use standardized protocols to delineate the core value and synergistic potential of individual adherence metrics.

This systematic review revealed that 83% (10/12) of eHealth interventions adopted multifunctional delivery modes, integrating auxiliary features, such as educational reminders, self-monitoring, and medication feedback (Tables S1 and S2 in Multimedia Appendix 2). Previous systematic reviews [7,62] suggest that complex intervention protocols may enhance medication adherence; however, our meta-analysis, constrained by limited sample size, failed to confirm this association (Figures S5 and S6 in Multimedia Appendix 3 and Table 2). Notably, while the COVID-19 pandemic presents an opportunity for the popularization of eHealth [63], it is uncertain whether adding too many metrics or features will be a barrier to long-term patient use. Reports from Norwegian nephrologists [64] indicate that while patients and health care providers encounter technical challenges, most endorse teleconsultation formats. Similar usability concerns emerged in this review, including practical barriers, such as device malfunction (3/12, 25%), data security concerns (2/12, 17%), and suboptimal user-device compatibility (2/17, 17%; Tables S1 and S2 in Multimedia Appendix 2). Therefore, systematic preimplementation assessments of patients’ perceived burdens and technological preferences are essential. Current barriers to research advancement include (1) scant empirical evidence on eHealth interventions’ impact on patient experience [65] and (2) the absence of process evaluations to identify effective components within complex intervention protocols [46].

High attrition rates in eHealth device use may compromise intervention efficacy. Han et al [38] reported a 11% (8/70) mobile app engagement rate at 6-month follow-up, while Mansell et al [41] observed 68.6% (120/175) completion of video education modules, findings consistent with reports by Low et al [37] regarding <40% MEMS use. This drop in participation is probably due to major problems in the technology design: 33% (4/12) of the programs did not focus on the needs of users, and the same percentage did not include feedback from health care professionals during their use (Table S1 in Multimedia Appendix 2). The analysis of 689 medical apps by Birkhoff et al [66] demonstrated that user-centered designs correlate with higher patient acceptance and clinical utility. A systematic review [67] indicated that sustained technology engagement is closely associated with perceived usefulness, ease of use, and self-efficacy. However, only 50% (6/12) of the included studies conducted device usability evaluations, and 25% (3/12) were limited to superficial satisfaction surveys. Furthermore, clinician involvement throughout the technology development process enhances the operationalization of interventions, where existing studies are significantly lacking. Current evidence suggests that limited patient co-design participation and lack of systematic usability assessments during trials may misalign eHealth functionalities with patient needs and values, driving suboptimal use rates and ultimately undermining outcome validity [68-71].

An alternative explanation for high attrition rates is “research fatigue” (2/12, 17%), wherein eHealth reminders demonstrate short-term efficacy but lose effectiveness as individuals’ sensitivity and responsiveness to stimuli diminish over time [40,42]. Although subgroup analyses suggested no apparent association between follow-up duration and adherence outcomes, this negative result may be related to inconsistent definitions of adherence across studies [72]. In addition, 50% (6/12) of the included studies had follow-up periods of 6 to 12 months, leaving long-term intervention effects beyond 12 months unestablished. Notably, Reese et al [42] found that interventions combining automated device alerts with clinician-delivered reminders significantly outperformed stand-alone device alerts in sustaining adherence (*P*<.05). This aligns with behavioral change theory’s core tenet: integrating external supports (eg, peer interactions and financial incentives) can strengthen intrinsic behavioral motivation [73-75]. However, only 50% (6/12) of interventions in this review were theoretically grounded in behavioral frameworks (Table S1 in Multimedia Appendix 2), revealing a theory-practice gap. We postulate that systematic integration of behavioral change theories into intervention design may enhance adherence sustainability, although empirical validation through robust clinical trials remains imperative.

The ceiling effect may also undermine outcome significance (4/12, 33%). Jung et al [11] showed that centralized monitoring systems based on information and communication technology did not help patients with a kidney transplant take their medication as prescribed because these patients already had very high adherence rates (99%-100%), a result supported by Low et al [37] and Foster et al [43]. Previous studies have circumvented this limitation through baseline assessments to deliver targeted interventions to nonadherent patients [76]. Future research should incorporate baseline screening to ensure successful eHealth device implementation among participants and exclude highly adherent populations at baseline.

Researchers appear cognizant that eHealth interventions may be preferentially suited for younger adult patients, who exhibit greater technological proficiency and demonstrate more observable engagement with digital tools. For instance, younger transplant recipients (aged <55 years) are significantly more likely to own smartphones (53/70, 75% vs 31/68, 46%; *P*<.001) and express willingness to adopt eHealth solutions (43/70, 62% vs 24/68, 36%; *P*=.02) [77]. Conversely, older patients appear to place higher value on face-to-face clinical interactions. A cross-sectional survey [78] found that many older patients were forced to use telemedicine as an alternative to face-to-face counseling during the COVID-19 pandemic but that most older patients were reluctant to continue using telemedicine even as the epidemic continued. This study provides a perspective on the psychological attitudes of older patients toward telemedicine. In addition, illiteracy (low health literacy) represents another critical barrier in eHealth research. Current studies frequently exclude illiterate transplant recipients, resulting in their underrepresentation in telemedicine literature [79,80]. Future investigations should address the unique needs of illiterate and older populations by developing tailored education or training protocols and implementing age-friendly, inclusive design strategies such as simplified interfaces, voice-guided prompts, and enlarged font sizes to reduce technological barriers.

### Future Research Directions and Recommendations

Although previous systematic reviews [76,81,82] have shown that various interventions can be effective in enhancing medication adherence in organ transplant recipients, these findings need to be considered in conjunction with the aforementioned confounding factors and interpreted with caution. Given the lack of high-quality evidence in this area, especially as rapid advances in information and communication technology and the global penetration of mobile devices continue to expand [83-85], we believe that eHealth interventions still have enormous potential.

This study underscores the need for higher-quality evidence to validate eHealth interventions’ efficacy in improving medication adherence among kidney transplant recipients. Key recommendations include the following:

1. Method standardization—future studies should adhere to standardized eHealth guidelines to harmonize outcome definitions and measurement tools, reducing methodological heterogeneity [84,86].
2. Methodological rigor—trial designs should be optimized using pilot data, baseline screening should be implemented to mitigate ceiling effects, and technology usability assessments and process evaluations should be conducted during implementation.
3. Theory integration—closed-loop intervention frameworks integrating behavioral theory should be developed (eg, “clinician-patient collaborative decision-making, real-time feedback, and adaptive intervention protocols”).
4. Risk transparency—transparent reporting of technology application risks (eg, data leakage and burden of use) is recommended.
5. Embracing null results—a positive view of negative or neutral results is essential for research quality improvement [87].
6. Quality strengthening—the risk of bias in this study was assessed as “some concerns” (11/12, 92%) and “high risk” (1/12, 8%), the main reason being inadequate implementation of blinding. A systematic review published in 2017 in the *Journal of the American Medical Association* [88] noted that when randomization and allocation concealment are rated as “low level,” trials can be considered high quality even if there are “some concerns” in other areas. However, the reliability of the results needs to be strengthened by adopting objective outcome indicators, standardizing the stratified randomization process, and expanding sample size and repeat validation. In this review, “some concerns” were caused by subjective self-reporting and the incomplete reporting of randomization details in some (4/12, 33%) studies. It is recommended that future studies adopt subjective and objective indicators and strictly follow the CONSORT (Consolidated Standards of Reporting Trials) statement for reporting randomization and allocation concealment details [89].
7. Research priorities—current evidence is limited by male participants who are young or middle aged, single-center, short follow-up, small sample sizes, and low to very low GRADE evidence quality ratings (Tables S1 and S5 in Multimedia Appendix 2). Urgently needed are more high-quality, multicenter RCTs with large samples (eg, older adults and illiterate people) and long-term follow-up to establish definitive clinical guidance.

### Strengths and Limitations

This study was based on 7 authoritative databases searched and included RCTs to strengthen the validity of the evidence. The Cochrane ROB 2 tool and the GRADE evidence grading system were used for quality assessment. The main limitations include the following: (1) Noninclusion of gray literature may have led to omission of evidence. (2) Only tacrolimus was selected as an indicator of immunosuppressive outcome (as it is the most commonly used drug after transplantation). (3) Some (7/12, 58%) studies did not provide analyzable data for inclusion in meta-analysis. (4) There were not enough studies to generate a funnel plot; however, this proportion is expected to be low, given that there have been trials that have published negative results. (5) The overall evidence is highly heterogeneous variability in adherence definitions, measurement tools, intervention modalities, and duration of follow-up.

Therefore, high-quality RCTs with large samples and standardized designs are needed to validate the long-term benefits of eHealth interventions.

### Conclusions

The evidence for this study is of low to very low quality. Owing to the small number of included studies and methodological heterogeneity, it is currently impossible to determine the efficacy of eHealth in promoting medication adherence in renal transplant recipients. In terms of research planning and implementation, eHealth still has considerable potential for improvement. However, we believe that eHealth remains a valuable tool for improving medication adherence. More high-quality, large-scale research is required to fully establish its efficacy.

## Appendix

Multimedia Appendix 1 PRISMA checklist.

Multimedia Appendix 2 Search strategy and other supplementary tables.

Multimedia Appendix 3 A summary of the risk of bias assessments and other supplementary figures.

## Abbreviations

BAASIS: Basel Assessment of Adherence to Immunosuppressive Medication Scale
CAS: Composite Adherence Score
CONSORT: Consolidated Standards of Reporting Trials
CV: coefficient of variation
GRADE: Grading of Recommendations, Assessment, Development, and Evaluation
IPV: intrapatient variability
MAM-MM: Medical Adherence Measure Medication Module
MD: mean difference
MEMS: Medication Event Monitoring System
PRISMA: Preferred Reporting Items for Systematic Reviews and Meta-Analyses
RCT: randomized controlled trial
ROB: Risk of Bias
RR: risk ratio
SMD: standardized mean difference
VAS: Visual Analog Scale
