# Supplementary material for: Effectiveness of eHealth for Medication Adherence in Renal Transplant Recipients: Systematic Review and Meta-Analysis
Source: J Med Internet Res. 2025 May 13;27:e73520. doi: 10.2196/73520 (PMC12117278; doi:10.2196/73520)
Supplement: Multimedia Appendix 2 [file jmir_v27i1e73520_app2.docx]

**Table S1(a-g).** Search strategy

**（a）**PubMed Search Trail (Search updated 19/11/2024)

| Search # | MeSH Terms and Key Words | Articles Revealed |
| --- | --- | --- |
| #1 | ("Kidney Transplantation"[Mesh]) OR (((Renal Transplant*) OR (Renal Kidney Transplant*)) OR (Kidney Transplantation)) | 197,988 |
| #2 | ((("Medication Adherence"[Mesh]) OR (((((((Medication Adherence) OR (Drug Adherence)) OR (Drug Compliance)) OR (Medication Persistence)) OR (Medication Nonadherence)) OR (Medication Noncompliance)) OR (Medication Compliance))) OR (Patient Compliance)) OR (Patient Adherence) | 256,451 |
| #3 | ((((((((((((((Telemedicine) OR (Mobile Health)) OR (mHealth)) OR (Telehealth)) OR (eHealth)) OR (Telecare)) OR (Virtual Medicine)) OR (Internet)) OR (software)) OR (website)) OR (telemanagement)) OR (telecommunications)) OR (telemonitoring)) OR ("Telemedicine"[Mesh])) OR (smartphone OR "cell phone" OR "mobile based" OR phone app OR "mobile app" OR "smartphone app" OR "mobile application" OR "self management" OR "cellular network" OR "self care" OR smartphone [MeSH] OR cell phone [MeSH] OR "mobile applications" [MeSH] OR internet [MeSH] OR self-management [MeSH]) | 884,416 |
| #4 | (((((Randomized Controlled Trial[Publication Type]) OR (Randomized Controlled Trial)) OR (Randomized)) OR (Randomised)) OR (Randomization)) OR (Randomisation) | 1,595,481 |
| #5 | #1 AND #2 AND #3 AND #4 | 51 |

**(b)**Web of Science Search Trail (Search updated 19/11/2024)

| Search # | Main Subjects and Key Words | Articles Revealed |
| --- | --- | --- |
| #1 | TS=((Renal Transplant*) OR (Renal Kidney Transplant*) OR (Kidney Transplantation)) and Preprint Citation Index (Exclude – Database) | 264,011 |
| #2 | TS=(Telemedicine OR (Mobile Health) OR mHealth OR Telehealth OR eHealth OR Telecare OR (Virtual Medicine) OR Internet OR software OR website OR telemanagement OR telecommunications OR telemonitoring OR smartphone OR (cell phone) OR (mobile based) OR (phone app) OR (mobile app) OR (smartphone app) OR (mobile application) OR (self management) OR (cellular network) OR (self care)) and Preprint Citation Index (Exclude – Database) | 2,515,860 |
| #3 | TS=((Medication Adherence) OR (Drug Adherence) OR (Drug Compliance) OR (Medication Persistence) OR (Medication Nonadherence) OR (Medication Noncompliance) OR (Medication Compliance) OR (Patient Compliance) OR (Patient Adherence)) and Preprint Citation Index (Exclude – Database) | 414,245 |
| #4 | TS=((Randomized Controlled Trial) OR Randomized OR Randomised OR Randomization OR Randomisation) and Preprint Citation Index (Exclude – Database) | 1,529,807 |
| #5 | #1 AND #2 AND #3 AND #4 | 167 |

**(c)** Cochrane Library Trail (Search updated 19/11/2024)

| Search # | MeSH Terms and Key Words | Articles Revealed |
| --- | --- | --- |
| #1 | (Renal Transplant*) OR (Renal Kidney Transplant*) OR (Kidney Transplantation) | 19,784 |
| #2 | Telemedicine OR (Mobile Health) OR mHealth OR Telehealth OR eHealth OR Telecare OR (Virtual Medicine) OR Internet OR software OR website OR telemanagement OR telecommunications OR telemonitoring OR smartphone OR (cell phone) OR (mobile based) OR (phone app) OR (mobile app) OR (smartphone app) OR (mobile application) OR (self management) OR (cellular network) OR (self care) | 155,222 |
| #3 | (Medication Adherence) OR (Drug Adherence) OR (Drug Compliance) OR (Medication Persistence) OR (Medication Nonadherence) OR (Medication Noncompliance) OR (Medication Compliance) OR (Patient Compliance) | 76,879 |
| #4 | (Randomized Controlled Trial) OR Randomized OR Randomised OR Randomization OR Randomisation | 1,331,761 |
| #5 | #1 AND #2 AND #3 AND #4 | 127 |

**(d)** Embase Trail (Search updated 19/11/2024)

| Search # | MeSH Terms and Key Words | Articles Revealed |
| --- | --- | --- |
| #1 | 'kidney transplantation'/exp OR 'kidney transplantation' OR 'kidney transplant' OR 'renal transplant' | 220,334 |
| #2 | 'medication adherence'/exp OR 'drug adherence' OR 'drug compliance' OR 'medication persistence' OR '' OR 'medication nonadherence' OR 'medication noncompliance' OR 'medication compliance' | 59,588 |
| #3 | 'telemedicine'/exp OR 'mobile health' OR mHealth OR telehealth OR eHealth OR telecare OR 'virtual medicine' OR internet OR software OR website OR telemanagement OR telecommunications OR telemonitoring OR smartphone OR 'cell phone' OR 'mobile based' OR 'phone app' OR 'mobile app' OR 'smartphone app' OR 'mobile application' OR 'self management' OR 'cellular network' OR 'self care' | 378,909 |
| #4 | 'randomized controlled trial'/exp OR 'randomized controlled trial' OR randomized OR randomised OR randomization OR randomisation | 1,673,015 |
| #5 | #1 AND #2 AND #3 AND #4 | 27 |

**(e)** CINAHL Search Trail (Search updated 19/11/2024)

| Search # | Subject Headings (MH) and Key Words | Articles Revealed |
| --- | --- | --- |
| #1 | (Renal Transplant*) OR (Renal Kidney Transplant*) OR (Kidney Transplantation) | 280,911 |
| #2 | Telemedicine OR (Mobile Health) OR mHealth OR Telehealth OR eHealth OR Telecare OR (Virtual Medicine) OR Internet OR software OR website OR telemanagement OR telecommunications OR telemonitoring OR smartphone OR (cell phone) OR (mobile based) OR (phone app) OR (mobile app) OR (smartphone app) OR (mobile application) OR (self management) OR (cellular network) OR (self care) | 12,936,768 |
| #3 | (Medication Adherence) OR (Drug Adherence) OR (Drug Compliance) OR (Medication Persistence) OR (Medication Nonadherence) OR (Medication Noncompliance) OR (Medication Compliance) OR (Patient Compliance) | 362,959 |
| #4 | (Randomized Controlled Trial) OR Randomized OR Randomised OR Randomization OR Randomisation | 2,521,668 |
| #5 | #1 AND #2 AND #3 AND #4 | 43 |

**(f) OVID** Search Trail (Search updated 19/11/2024)

| Search # | Subject Headings (MH) and Key Words | Articles Revealed |
| --- | --- | --- |
| #1 | (Renal Transplant*) OR (Renal Kidney Transplant*) OR (Kidney Transplantation) | 125,983 |
| #2 | Telemedicine OR (Mobile Health) OR mHealth OR Telehealth OR eHealth OR Telecare OR (Virtual Medicine) OR Internet OR software OR website OR telemanagement OR telecommunications OR telemonitoring OR smartphone OR (cell phone) OR (mobile based) OR (phone app) OR (mobile app) OR (smartphone app) OR (mobile application) OR (self management) OR (cellular network) OR (self care) | 671,446 |
| #3 | (Medication Adherence) OR (Drug Adherence) OR (Drug Compliance) OR (Medication Persistence) OR (Medication Nonadherence) OR (Medication Noncompliance) OR (Medication Compliance) OR (Patient Compliance) | 106,895 |
| #4 | (Randomized Controlled Trial) OR Randomized OR Randomised OR Randomization OR Randomisation | 1,175,145 |
| #5 | #1 AND #2 AND #3 AND #4 | 26 |

**(g) SCOPUS** Search Trail (Search updated 19/11/2024)

| Search # | Subject Headings (MH) and Key Words | Articles Revealed |
| --- | --- | --- |
| #1 | (Renal Transplant*) OR (Renal Kidney Transplant*) OR (Kidney Transplantation) | 343,9160 |
| #2 | Telemedicine OR (Mobile Health) OR mHealth OR Telehealth OR eHealth OR Telecare OR (Virtual Medicine) OR Internet OR software OR website OR telemanagement OR telecommunications OR telemonitoring OR smartphone OR (cell phone) OR (mobile based) OR (phone app) OR (mobile app) OR (smartphone app) OR (mobile application) OR (self management) OR (cellular network) OR (self care) | 789,449 |
| #3 | (Medication Adherence) OR (Drug Adherence) OR (Drug Compliance) OR (Medication Persistence) OR (Medication Nonadherence) OR (Medication Noncompliance) OR (Medication Compliance) OR (Patient Compliance) | 327,314 |
| #4 | (Randomized Controlled Trial) OR Randomized OR Randomised OR Randomization OR Randomisation | 5,849,860 |
| #5 | #1 AND #2 AND #3 AND #4 | 648 |

**Table S2. Quantitative statistics on study characteristics (n=12)**

| **Characteristic** | **n(%)** |
| --- | --- |
| **Sex** |  |
| Male ≥50% | 11(92) |
| Unspecified | 1(9) |
| **Age (mean or median)** |  |
| 40-60 | 11(92) |
| <18 | 1(8) |
| **Publication year** |  |
| 2015-present | 11(92) |
| 2013 | 1(8) |
| **Country** |  |
| United States of America | 4(33) |
| Turkey | 1(8) |
| Australia | 1(8) |
| South Korea | 2(17) |
| Germany | 1(8) |
| Sweden | 1(8) |
| Canada | 2(17) |
| **Number of participants** |  |
| 0-99 | 5(42) |
| 100-199 | 7(58) |
| **Duration of follow-up (months)** |  |
| ≤ 3 | 2(17) |
| >3 to <6 | 4(33) |
| ≥12 | 6(50) |
| **Mode of ehealth delivery** |  |
| Mixed mode (>1mode of eHealth delivery) | 10(83) |
| Single mode | 2(17) |
| **Functionality/intervention purpose （Ehealth function）** |  |
| Multifunctional | 10(38) |
| Single function | 2(17) |
| **Intervention is based on behavioral theory** |  |
| Theory based | 6(50) |
| No theory based or not reported | 6(50) |
| **User centered design** |  |
| Patient involvement | 8(67) |
| No patient involvement or not reported | 4(33) |
| **Usability assessment for ehealth** |  |
| Evaluated for user satisfaction | 3(25) |
| Evaluated for user understanding | 1(8) |
| Evaluated for user attitude or beliefs | 2(17) |
| Not reported | 6(50) |
| **Clinician participation** |  |
| Yes | 8(67) |
| No | 4(33) |
| **Adherence measurement method** |  |
| Self-reporting + Electronic testing +Tacrolimus Biochemical Indicators | 1(8) |
| Self-reporting + Electronic testing | 3(25) |
| Electronic testing +Tacrolimus Biochemical Indicators | 3(25) |
| Self-reporting+Tacrolimus Biochemical Indicators | 2(17) |
| Tacrolimus Biochemical Indicators （TAC blood concentration + TAC-IPV + TAC-CV＜40%） | 1(8) |
| Electronic testing | 1(8) |
| Other(Physician assessment + self-report + Tacrolimus Biochemical Indicators | 1(8) |
| **Major concerns (user level)** |  |
| Malfunction of electronic equipment | 3(25) |
| Unstable network signal | 1(8) |
| Time constraints/too busy/travel constraints | 3(25) |
| Electronic devices too heavy | 1(8) |
| Not interested/disliked | 2(17) |
| Electronic devices make people feel uncomfortable/surveillance/stressful | 2(17) |
| Difficult to integrate with daily medication habits/not applicable | 2(17) |
| Data insecurity/privacy | 2(17) |
| **Other Concerns** |  |
| High attrition rate (low use of electronic devices/willful non-compliance) | 4(33) |
| Electronic equipment: high cost of time, labor, technology/reimbursement by health insurance | 4(33) |
| Accuracy of electronic pillbox monitoring | 2(17) |
| Self-reported vulnerability to bias (e.g., recall bias, white coat effect) | 2(17) |
| Blood levels are susceptible to many factors | 4(33) |
| Ceiling effect (inclusion of high adherence participants at baseline) | 4(33) |
| Research fatigue (lack of incentives to persist over time) | 2(17) |
| Single center | 7(58) |
| Multicenter | 3(25) |
| Single (multi) center not reported | 2(17) |

Footnotes:

TAC= tacrolimus; TAC-IPV = Intra-patient variability of tacrolimus concentrations; TAC-CV= tacrolimus coefficient of variation; TAC-CV< 40% = Proportion of patients with tacrolimus coefficient of variation <40%.

**Table S3. Additional information on study characteristics**

| **First**  **author** | **Male n(%)** | **Theory** | **Ehealth delivery** | **Human involvement (User centered design)** | **Usability**  **assessment** | **Major concerns**  **(user level)** |
| --- | --- | --- | --- | --- | --- | --- |
|  |  |  | **Ehealth function** |  |  | **Other concerns** |
| Erdal et al. 2025 | 79  (79) | NR | Text message on cell phone | NH | Small-scale pilot to assess program comprehension | NR |
|  |  |  | Educational, Reminder |  |  | 1.Single center  2.Blood levels are susceptible to many factors |
| McGillicuddy et al. 2013 | 11  (57.9) | Self determination theory | Electronic drug tray，SMS, phone or email alerts | The researcher texted, emailed, or contacted individuals when electronic warnings suggested medication noncompliance. **Physicians** modified the regimen weekly using electronic records, alerting **study investigators** and **patients** via email. | A quantitative assessment of participants' intervention attitudes before the research. | 1 participant experienced equipment failure;  8 participants feared limited travel, busyness, or heavy medication trays; 6 participants considered themselves highly compliant;  5 participants had weak network signal. |
|  |  |  | Reminder,  Self monitoring and feedback |  |  | 1.Cost: $45 per month  2.Self-reporting overestimates adherence  3.Single center |
| Fleming et al.  2021 | 77  (56.6) | NR | APP, smartphone | The TRANSAFE Rx app reminds patients to take their meds and generates monthly **pharmacist** and **nurse** feedback. | A post-study satisfaction survey | NR |
|  |  |  | Reminder,  Self monitoring and feedback |  |  | 1.Single center  2.Blood levels are susceptible to many factors  3.Ceiling effect |
| McGillicuddy et al. 2020 | 57  (69) | Self determination theory | Electronic medication tray, smartphone | Electronic medication trays, phone calls or tailored incentive text messages to remind **patients** to take their pills, and biweekly **healthcare professional** medication administration evaluations. | Participants' values, beliefs, and goals were quantified at baseline | NR |
|  |  |  | Reminder,  Self monitoring and feedback |  |  | 1.Single center  2.Short follow-up time |
| Low et al.2019 | 41  (57.7) | Planned behavior theory | Electronic video, cell phone,  electronic pillbox (monitoring only) | A medical team  (**1 kidney donor, 2 general practitioners, 5 healthcare workers**) meets biweekly for medication assessments, consumer-centered videos, and health coaching | NR | 1 participant experienced equipment failure;  3 participants lost their electronic pill bottles (1 voluntarily discarded);  1 participant did not use the electronic pill bottle |
|  |  |  | Educational, Reminder,  Self monitoring and feedback, Behavioral counseling |  |  | 1.High attrition rate:only <40% adhere to electronic monitors  2.Ceiling effect  3.Multicenter |
| Han et al. 2019 | 88  (64.7) | NR | Adhere4U app (mobile phone)  ,Electronic pillbox (monitoring only) | NH | NR | 9 Participants' e-med bottle was lost or damaged;  13 Participant feeling uncomfortable |
|  |  |  | Educational, Reminder,  Self monitoring and feedback, |  |  | 1.High attrition rate:App usage was only 12% at six months  2. only patients ≥1 year post-transplantation were included  3. Single center |
| Schmid et al.  2017 | 14  (61) | NR | Telehealth Monitoring System (laptop, cell phone) | A transplant **nurse** case manager and two senior transplant physicians (**nephrologist and surgeon**) can remotely monitor and provide real-time video-screen consultations using telemedicine. | NR | NR |
|  |  |  | Educational, Reminder,  Self monitoring and feedback, Behavioral counseling |  |  | 1. requires additional staff (50% part-time transplant nurses), but is cost-effective  2. No standard reimbursement  3. Limited to living donor kidney recipients only  4. Data protection laws prohibit the use of tablets with mobile remote monitoring software  5. Single center |
| Henriksson et al.2016 | Unspecified | NR | Web-based electronic medication dispenser | NH | NR | 3 participants felt 'monitored';  1 participant felt stressed by the device |
|  |  |  | Reminder |  |  | 1.Research fatigue :lack of incentives to persist over time  2. Single center |
| Mansell et al.  2024 | 110  (63.6) | Behavioral motivation theory | Electronic video | **Participants** were required to watch a medication education video (in the hospital or at home) | NR | NR |
|  |  |  | Educational |  |  | 1. High attrition rate: only 69% of participants persisted in completion, possibly related to willful non-compliance  2. Electronic monitoring is costly and time-consuming  3. No data security monitoring committee  4. Multi-center |
| Reese et al.  2017 | 72  (60) | Behavioral  economic theory | Electronic monitoring pillbox  , mobile phone | If medication adherence in Intervention Group 1 is less than 90%, the **study coordinator** and **nephrologist** will contact the **patient** by phone | Qualitative results showed many participants welcomed electronic pill bottle reminders. | Reported difficulty integrating wireless pill bottles into their medication-taking routines. |
|  |  |  | Reminder,  Self monitoring and feedback, |  |  | 1.Research fatigue :lack of incentives to persist over time  2.Accuracy of electronic pillbox monitoring  3.Blood levels are susceptible to many factors  4.Single (multi) center not reported |
| Jung et al.2020 | 60  (57.1) | NR | The ICT: a smart pill box , home monitoring systems,eCRF, and CTMS . | Text and pillbox warnings notify **patients** and **medical staff** of dosage, administration timing errors and missed doses. | Quantitative survey of participants' satisfaction with ICT (at 4 and 24 weeks) | 1 participant feels insecure;  1 participant felt time constrained;  5 participants found maladjustment to the system (not applicable) |
|  |  |  | Reminder,  Self monitoring and feedback, |  |  | 1. Ceiling effect: high baseline adherence  2.Accuracy of electronic pillbox monitoring  3.Network stability  4. Single (multi) center not reported |
| Foster et al.  2018 | 54  (61) | Selfmanagement  model | Electronic monitoring pillbox  (Medminder or SimpleMed) ,  SMS, email, or visual signals | A quarterly adherence  support group of patients, coaches (**Psychologist, nurse**)and parent jointly reviews medication adherence data and identifies barriers to use. | NR | 51 out of 105 refused consent are not interested.  21 participants didn't like pillbox, 23 participants didn't have the time/were too busy, the  1 participant was concerned about privacy  During intervention interval: 4 participants didn't like pillbox, 1 participant didn't have time for it |
|  |  |  | Educational, Reminder,  Self monitoring and feedback, Behavioral counseling |  |  | 1.Self-reporting is vulnerable to multiple biases ('recall bias,' 'underreporting bias,' 'white coat effect')  2. Electronic monitoring devices: high time, labor, and technology costs  3. Blood levels are susceptible to multiple factors  4.Ceiling effect  5. Multi-center |

Footnotes:

NR= Not reported; NH= No human involvement; SMS= Short Message Service,; APP=Application; ICT= the information and communication technology; eCRF: a home monitoring system; CTMS= Integrated Clinical Trials Management System.

**Table S4. Summary of quantitative/qualitative findings**

| **Analytical method** | **Name** | **Data type** | **Results (Calculated where sufficient data reported or *p* values , [95% confidence interval])** |
| --- | --- | --- | --- |
| **Medication adherence assessed by self-report** | | | |
| **Meta-**  **analysis** | Mansell et al. (2024，Canada) | Dichotomized data | 0.85 [0.63,1.14] |
|  | Han et al. (2019，South Korea) | Dichotomized data | 1.30 [0.89,1.89] |
|  | Reese et al. (2017,USA)^(1)^ | Dichotomized data | 0.91 [0.72,1.16] |
|  | Reese et al. (2017,USA)^(2)^ | Dichotomized data | 0.95 [0.76,1.19] |
|  | **Meta summary result** | | **0.98[0.85, 1.13]** |
|  | Erdal et al. (2024,Turkey) | Continuous data | 0.75 [0.35,1.16] |
|  | Foster et al. (2018,Canada)^(3)^ | Continuous data | 0.22 [-0.08,0.53] |
|  | Foster et al. (2018,Canada)^(4)^ | Continuous data | 0.24 [-0.07,0.55] |
|  | **Meta summary result** | | **0.38[0.07,0.68]*** |
| **Narrative synthesis** | Low et al. (2019,Australia) | Specific values not given, not suitable for meta-analysis | From baseline to 12 months, the control group had a significantly lower rate of medication adherence compared to the intervention group (*P*<.001)* |
| **Medication adherence monitored by electronic devices** | | | |
| **Meta-**  **analysis** | Han et al. (2019，South Korea) | dichotomized data | 0.92 [0.57,1.49] |
|  | McGillicuddy et al. (2020，USA) | dichotomized data | 1.96 [1.35,2.86] |
|  | Reese et al. (2017,USA)^(1)^ | dichotomized data | 1.58 [1.16,2.15] |
|  | Reese et al. (2017,USA)^(2)^ | dichotomized data | 1.40 [1.01,1.95] |
|  | **Meta summary result** | | **1.46[1.11,1.90]*** |
|  | McGillicuddy et al. (2013,USA) | continuous data | 0.37 [0.27, 0.47] |
|  | **Meta summary result** | | **0.37 [0.27, 0.47]*** |
| **Narrative synthesis** | Low et al. (2019,Australia) | Specific values not given, not suitable for meta-analysis | It assessed that the effect was comparable between the two groups |
|  | Henriksson et al. (2016,Sweden) | Specific values not given, not suitable for meta-analysis | Medication adherence in the intervention group was reported at 97.8%, while the control group was not monitored |
|  | Jung et al. (2020,South Korea) | Specific values not given, not suitable for meta-analysis | Dose-taking adherence was >98% in both groups, and there were no significant differences in dose-frequency adherence, dose-interval adherence, or medication holiday |
|  | Foster et al. (2018,Canada) | Specific values not given, not suitable for meta-analysis | OR=1.66, 95% CI (1.15, 2.39), *P*=.006^(3)*^  OR=1.74, 95% CI (1.21, 2.50), *P*=.003^(4)^* |
| **Tacrolimus Blood Concentration** | | | |
| **Meta-**  **analysis** | Reese et al. (2017,USA)^(1)^ | continuous data | -0.30 [-1.02,0.42] |
|  | Reese et al. (2017,USA)^(2)^ | continuous data | 0.32 [-0.67,1.31] |
|  | Jung et al. (2020,South Korea) | continuous data | 0.30 [-0.16,0.76] |
|  | **Meta summary result** | | **0.15[-0.21,0.51]** |
|  | Erdal et al. (2024,Turkey) | dichotomized data | 1.38 [1.04,1.82] |
|  | **Meta summary result** | | **1.38 [1.04,1.82]*** |
| **Narrative synthesis** | Henriksson et al. (2016,Sweden) | Specific values not given, not suitable for meta-analysis | No significant difference in both control and intervention groups |
| **Intra-patient variability of tacrolimus concentrations** | | | |
| **Meta-**  **analysis** | Reese et al. (2017,USA)^(1)^ | continuous data | -0.03 [-0.10,0.04] |
|  | Reese et al. (2017,USA)^(2)^ | continuous data | -0.01 [-0.08,0.06] |
|  | Jung et al. (2020,South Korea) | continuous data | -1.20 [-5.99,3.59] |
|  | **Meta summary result** | | **-0.02[-0.07, 0.03]** |
| **Narrative synthesis** | Fleming et al. (2021,USA) | Specific values not given, not suitable for meta-analysis | Significant difference between control and intervention groups (*P*=.0133)* |
|  | McGillicuddy et al. (2020，USA) | Specific values not given, not suitable for meta-analysis | Significant difference between control and intervention groups (*P*=.046)* |
|  | Mansell et al. (2024，Canada) | Specific values not given, not suitable for meta-analysis | Did not exhibit a statistically significant difference between the two groups, with the 95% CI(-0.018, 0.071) |
| **Proportion of patients with tacrolimus coefficient of variation <40%** | | | |
| **Narrative synthesis** | Fleming et al. (2021,USA) | Specific values not given, not suitable for meta-analysis | No significant difference in both control and intervention groups (*P*= .224). However, the proportion of patients with tacrolimus <30% was significantly different between the two groups (*P*=.033), related to the ceiling effect |
|  | McGillicuddy et al. (2020，USA) | Specific values not given, not suitable for meta-analysis | The intervention group had a higher tacrolimus CV < 40% after a 6-month randomized controlled trial (80% versus 70%, *P*=.001)* |
| **Comprehensive scoring of medication adherence (subjective plus objective).** | | | |
| **Meta-**  **analysis** | Schmid et al. (2017,Germany) | dichotomized data | 1.90 [1.15,3.14] |
|  | **Meta summary result** | | **1.90 [1.15,3.14]*** |

Footnotes:

1. Group1: medication reminders from doctors and wireless pill bottles; (2) Group2: medication reminders from wireless pill bottles; (3) Taking adherence; (4) Timing adherence; CV= coefficient of variation ;CI= confidence interval;*=Significant difference between control and intervention groups

**Table S5. Summary of sensitivity analysis**

| **Excluded variable** | **Original result** | | | | **Results after exclusion** | | **Changes in results** |
| --- | --- | --- | --- | --- | --- | --- | --- |
|  | **RR/SMD 95%CI, *P*** | | | **Heterogrnrity**  ***P，I^2^*** | **RR/SMD 95%CI, *P*** | **Heterogrnrity**  ***I^2^，P*** |  |
| **Adherence rate by different assessment method** | | | | | | | |
| **Self-report (dichotomized data)** | | | | | | | |
| **-** | 0.98[0.85,1.13], *P*=.73 | *P*=.09, *I^2^*=59% | | | - | | **-** |
| 9.Mansell et al. (2024，Canada) | 0.98[0.85,1.13], *P*=.73 | *P*=.09, *I^2^*=59% | | | 1.03[0.88,1.21],  *P*=.72 | *P*=.23*, I^2^*=32% | - |
| 6.Han et al. (2019，South Korea) | 0.98[0.85,1.13], *P*=.73 | *P*=.09, *I^2^*=59% | | | 0.90 [0.77,1.05], *P*=.17; | *P*= .83*, I^2^*=0% | - |
| 10.Reese et al. (2017,USA)^(1)^ | 0.98[0.85,1.13], *P*=.73 | *P*=.09, *I^2^*=59% | | | 1.00 [0.84,1.18], *P*=.97 | *P*=.21, *I^2^*=36% | - |
| 10.Reese et al. (2017,USA)^(2)^ | 0.98[0.85,1.13], *P*=.73 | *P*=.09, *I^2^*=59% | | | 0.98 [0.83,1.17], *P*=.86 | *P*=.18*, I^2^*=41% | - |
| **Self-report (continuous data)** | | | | | | | |
| **-** | 0.38[0.07,0.68], *P*=.01 | | *P*=.09*, I^2^*=59% | | **-** | | **-** |
| 1.Erdal et al. (2024,Turkey) | 0.38[0.07,0.68], *P*=.01 | | *P*=.09*, I^2^*=59% | | 0.23[0.01,0.45], *P*=.04 | *P*=.94, *I^2^*=0% | - |
| 12.Foster et al. (2018,Canada)^(3)^ | 0.38[0.07,0.68], *P*=.01 | | *P*=.09*, I^2^*=59% | | 0.48[-0.02,0.98], *P*=.06 | *P*=.05, *I^2^*=74% | ***** |
| 12.Foster et al. (2018,Canada)^(4)^ | 0.38[0.07,0.68], *P*=.01 | | *P*=.09*, I^2^*=59% | | 0.47[-0.05,0.9] *P*=.07 | *P*=.04, *I^2^*=76% | ***** |
| Footnotes:  *= Significant changes in results   1. Group 1: medication reminders from doctors and wireless pill bottles 2. Group 2: medication reminders from wireless pill bottles 3. Taking adherence 4. Timing adherence | | | | | | | |
| **Electronic monitoring (dichotomized data)** | | | | | | | |
| - | 1.46[1.11,11.90],*P*=.006 | | *P*=.09, *I^2^*=53% | | - | | - |
| 6.Han et al.(2019，South Korea) | 1.46[1.11,11.90],*P*=.006 | | *P*=.09, *I^2^*=53% | | 1.61[1.32,1.95],*P*<.00001 | *P*=.41*, I^2^*=0% | - |
| 4.McGillicuddy et al. (2020，USA) | 1.46[1.11,11.90],*P*=.006 | | *P*=.09, *I^2^*=53% | | 1.33[1.00,1.77], *P*=.05 | *P*=.16, *I^2^*=46% | * |
| 10.Reese et al. (2017,USA)^(1)^ | 1.46[1.11,11.90],*P*=.006 | | *P*=.09, *I^2^*=53% | | 1.40[0.94,2.07], *P*=.10 | *P*=.05, *I^2^*=68% | * |
| 10.Reese et al. (2017,USA)^(2)^ | 1.46[1.11,11.90],*P*=.006 | | *P*=.09, *I^2^*=53% | | 1.46[0.98,2.16], *P*=.06 | *P*=.04, *I^2^*=68% | * |
| **Electronic monitoring (continuous data)** | | | | | | | |
| - | 0.37 [0.27, 0.47],*P*<.0001 | | | | - | | - |
| 2.McGillicuddy et al. (2013,USA) | 0.37 [0.27, 0.47],*P*<.0001 | | | | - | | - |
| Footnotes:  *= Significant changes in results   1. Group 1: medication reminders from doctors and wireless pill bottles 2. Group 2: medication reminders from wireless pill bottles | | | | | | | |
| **Tacrolimus Blood Concentration (dichotomized data)** | | | | | | | |
| - | 1.38[1.04,1.82],*P=*.02 | | | | - | | - |
| 1.Erdal et al.(2025,Turkey) | 1.38[1.04,1.82],*P=*.02 | | | | - | | - |
| **Tacrolimus Blood Concentration (continuous data)** | | | | | | | |
| - | 0.15[-0.21,0.51], *P*=.41 | | *P*=.37, *I^2^*=1% | | - | | - |
| 10.Reese et al. (2017,USA)^(1)^ | 0.15[-0.21,0.51], *P*=.41 | | *P*=.37, *I^2^*=1% | | 0.30[-0.11,0.72], *P*=.15 | *P*=.97, *I^2^*=0% | - |
| 10.Reese et al. (2017,USA)^(2)^ | 0.15[-0.21,0.51], *P*=.41 | | *P*=.17, *I^2^*=47% | | 0.13[-0.26,0.51], *P*=.52 | *P*=.15, *I^2^*=51% | - |
| 11.Jung et al.(2020,South Korea) | 0.15[-0.21,0.51], *P*=.41 | | *P*=.37, *I^2^*=1% | | -0.08[-0.67,0.50], *P*=.78 | *P*=.78*, I^2^*=0% | - |
| Footnotes:  *= Significant changes in results   1. Group 1: medication reminders from doctors and wireless pill bottles 2. Group 2: medication reminders from wireless pill bottles | | | | | | | |
| **Intra-patient variability of tacrolimus concentrations (continuous data)** | | | | | | | |
| - | -0.02[-0.07,0.03],*P*=.40 | | *P*=.82, *I^2^*=0% | | - | | - |
| 10.Reese et al. (2017,USA)^(1)^ | -0.02[-0.07,0.03],*P*=.40 | | *P*=.82, *I^2^*=0% | | -0.01[-0.08,0.06],*P*=.78 | *P*=.63, *I^2^*=0% | - |
| 10.Reese et al. (2017,USA)^(2)^ | -0.02[-0.07,0.03],*P*=.40 | | *P*=.82, *I^2^*=0% | | -0.03[-0.10,0.04],*P*=.38 | *P*=.63, *I^2^*=0% | - |
| 11.Jung et al.(2020,South Korea) | -0.02[-0.07,0.03],*P*=.40 | | *P*=.82, *I^2^*=0% | | -0.02[-0.07,0.03],*P*=.41 | *P*=.69, *I^2^*=0% | - |
| Footnotes:  *= Significant changes in results   1. Group 1: medication reminders from doctors and wireless pill bottles 2. Group 2: medication reminders from wireless pill bottles | | | | | | | |
| **Comprehensive scoring (dichotomized data)** | | | | | | | |
| - | 1.90 [1.15, 3.14], *P*=.01 | | | | - | | - |
| 7.Schmid et al.(2017,Germany) | 1.90 [1.15, 3.14], *P*=.01 | | | | - | | - |

**Table S6.** **Assessment of quality of trials using the Grading of Recommendations, Assessment, Development and Evaluation (GRADE) system ---------- Outcome indicators to assess medication adherence**

| **Number of trials** | **Participants, n** | **Risk of bias** | **Inconsistency** | **Indirectness** | **Imprecise** | **Publication bias** | **Quality** |
| --- | --- | --- | --- | --- | --- | --- | --- |
| **Self-reporting assessment** | | | | | | | |
| 6RCTs^a^ | 643 | Serious ^b^  Unclear allocation concealment^(1)^  No blinding possible ^(2)^ | Serious ^c^  Inconsistency of results  *I^2^* = 59%, *P*= .09  Differences in  eHealth intervention  and standard of care  Differences in trial duration | Not serious | Not serious | Unable to be determined | ⊕⊕⊖⊖  low |
| **Electronic device monitoring** | | | | | | | |
| 8RCTs^a^ | 809 | Serious ^b^  Incomplete reporting of random sequence generation^(3)^  Unclear allocation concealment^(4)^  No blinding possible^(5^ | Serious ^c^  Inconsistency of results  *I^2^* = 53%, *P*= .09  Differences in  eHealth intervention  and standard of care  Differences in trial  duration | Not serious | Not serious | Unable to be determined | ⊕⊕⊖⊖  low |
| **Tacrolimus blood concentrations** | | | | | | | |
| 4RCTs^a^ | 402 | Serious ^b^  No blinding possible^(6)^  Unclear allocation concealment^(7)^ | Serious ^c^  Inconsistency of results  Differences in  eHealth intervention  and standard of care  Differences in trial  duration | Not serious | Not serious | Unable to be determined | ⊕⊕⊖⊖  low |
| **Intra-patient variability of tacrolimus concentrations** | | | | | | | |
| 5RCTs^a^ | 613 | Serious ^b^  Incomplete reporting of random sequence generation^(8)^  Unclear allocation concealment^(8)^  No blinding possible^(9)^ | Serious ^c^  Inconsistency of results  Differences in  eHealth intervention  and standard of care  Differences in trial  duration | Not serious | Not serious | Unable to be  determined | ⊕⊕⊖⊖  low |
| **Tacrolimus coefficient of variation <40%** | | | | | | | |
| 2RCTs^a^ | 218 | Serious ^b^  Incomplete reporting of random sequence generation^(10)^  Unclear allocation concealment^(10)^  No blinding possible^(10)^ | Serious ^c^  Inconsistency of results  Differences in  eHealth intervention  and standard of care  Differences in trial  duration | Not serious | Serious ^d^ | Unable to be  determined | ⊕⊖⊖⊖  Very  low |
| **A composite adherence score (CAS)** | | | | | | | |
| 1RCT^a^ | 46 | Serious ^e^  No blinding possible^(11)^ | Not serious | Not serious | Serious ^d^ | Unable to be  determined | ⊕⊕⊖⊖  Low |

Footnotes:

^a^RCT: randomized controlled trial.

^b^Downgrade due to the majority of trials rated as some concerns.

^c^Downgrade due to I^2^ statistics >50%, inconsistency of results, differences in eHealth intervention and standard of care, or differences in trial duration.

^d^Downgrade due to pooled sample sizes <400.

^e^ Downgrade due to the non-use of blinding of assessors.

Reference (the literature)

(1)Foster et al., 2018

(2)Erdal et al., 2015; Low et al., 2019; Han et al., 2019; Mansell et al., 2024; Reese et al., 2017; Foster et al., 2018

(3)McGillicuddy et al., 2013; McGillicuddy et al., 2020; Henriksson et al., 2016

(4)McGillicuddy et al., 2013; McGillicuddy et al., 2020; Foster et al., 2018

(5)McGillicuddy et al., 2013; McGillicuddy et al., 2020; Low et al., 2019; Han et al., 2019; Henriksson et al., 2016; Reese et al., 2017; Jung et al., 2020; Foster et al., 2018

(6)Erdal et al., 2015; Henriksson et al., 2016; Reese et al., 2017; Jung et al., 2020

(7) Henriksson et al., 2016

(8)Fleming et al., 2021; McGillicuddy et al., 2020

(9)Fleming et al., 2021; McGillicuddy et al., 2020; Mansell et al., 2024; Reese et al., 2017; Jung et al., 2020

(10) Fleming et al., 2021; McGillicuddy et al., 2020

(11)Schmid et al., 2017

GRADE Working Group grades of evidence: 1) High quality: Further research is very unlikely to change our confidence in the estimate of effect. 2) Moderate quality: Further research is likely to have an important impact on our confidence in the estimate of effect and may change the estimate. 3) Low quality: Further research is very likely to have an important impact on our confidence in the estimate of effect and is likely to change the estimate. 4) Very low quality: We are very uncertain about the estimate.
