# Supplementary material for: Effectiveness of eHealth for Medication Adherence in Renal Transplant Recipients: Systematic Review and Meta-Analysis
Source: J Med Internet Res. 2025 May 13;27:e73520. doi: 10.2196/73520 (PMC12117278; doi:10.2196/73520)
Supplement: Multimedia Appendix 3 [file jmir_v27i1e73520_app3.docx]

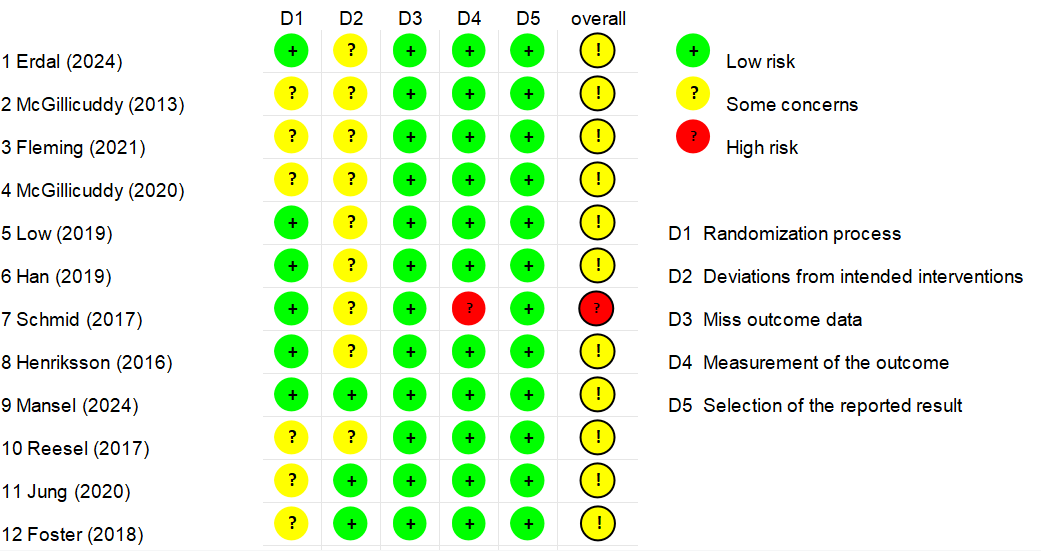


**Fig. S1** Cochrane Risk of Bias tool for randomized trials (RoB 2.0).


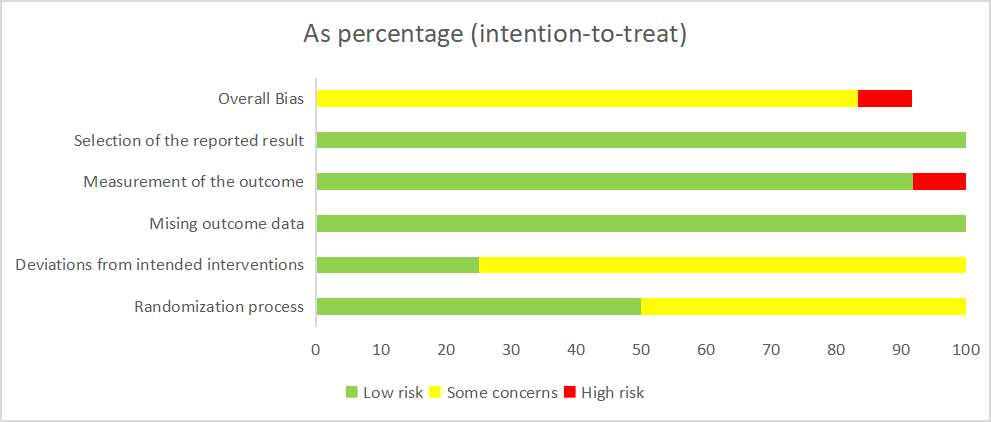


**Fig. S2** Summary of bias risk of included studies.

**(A)**


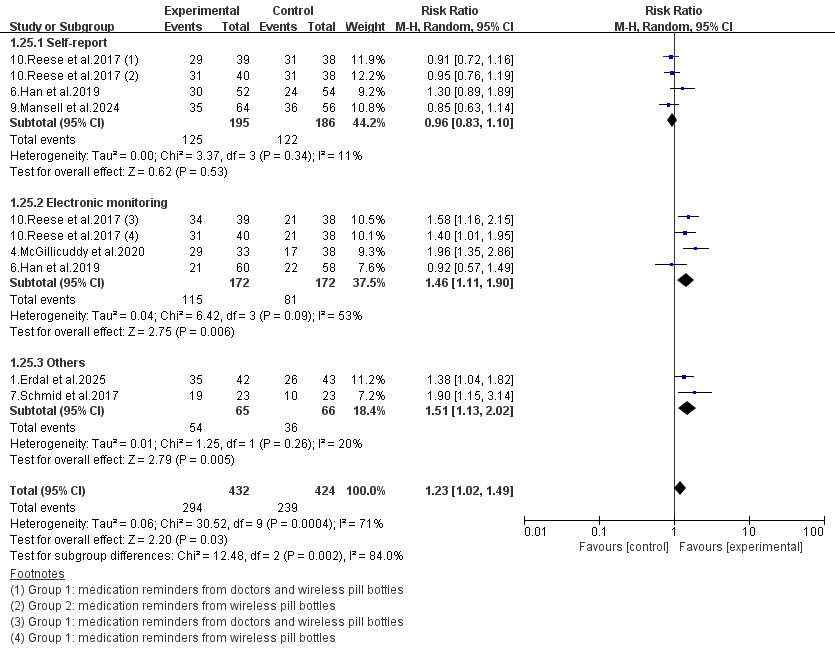


**(B)**


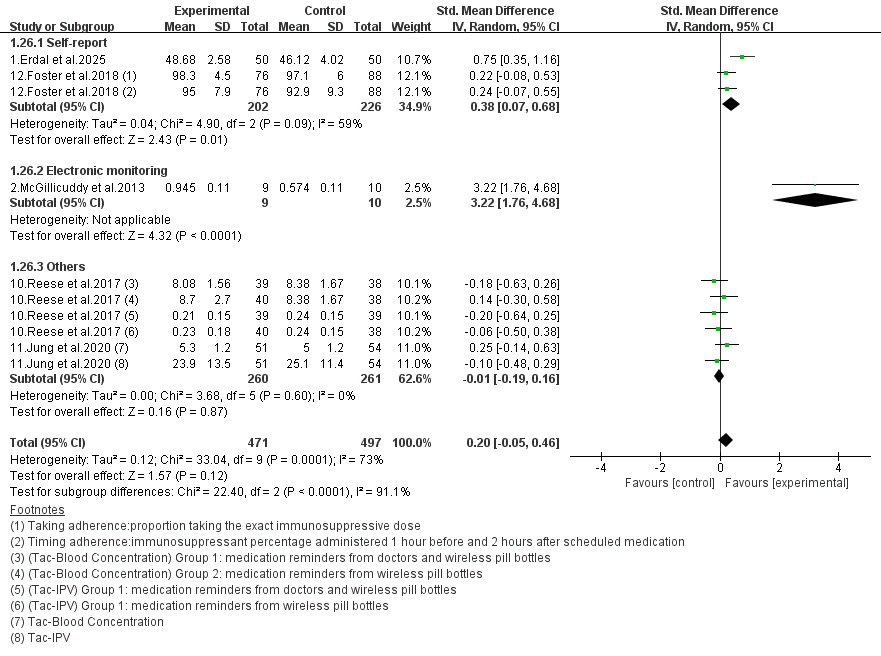


**Fig. S3 Subgroup by adherence assessment methods: (A) dichotomized data, (B) continuous data.**

**（A）**

**
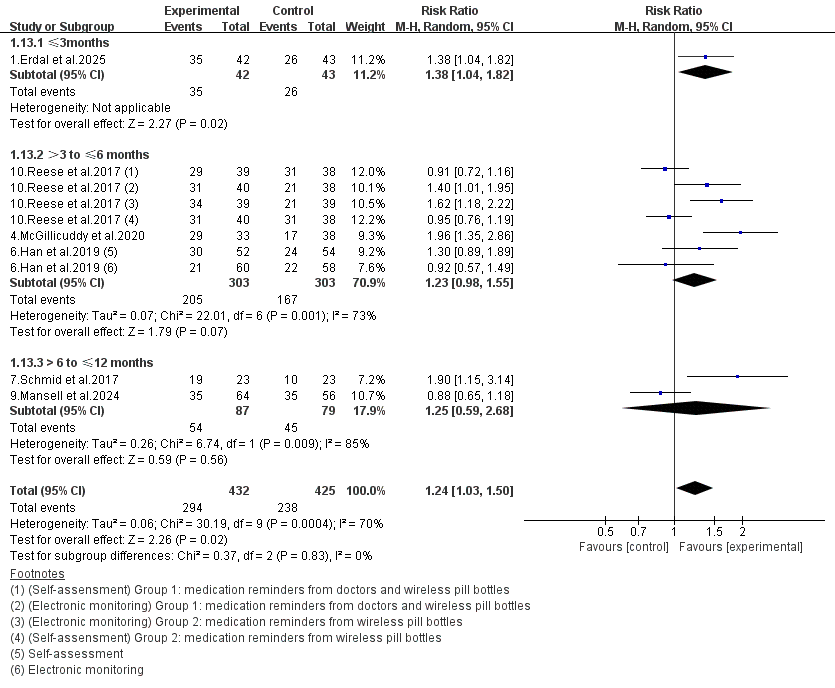
**

**(B)**

**
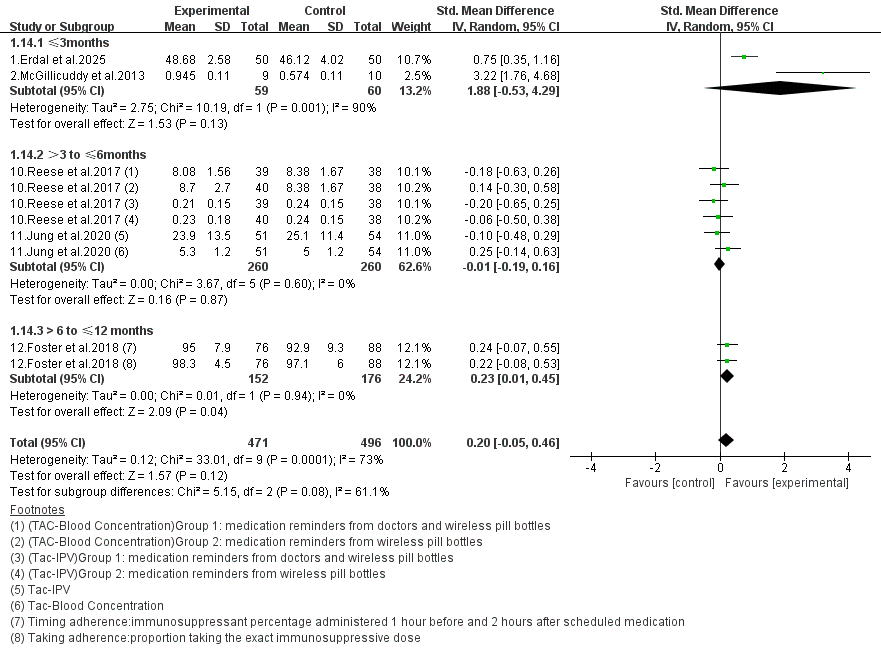
**

**Fig. S4 Subgroup by follow-up time: (A) dichotomized data, (B) continuous data.**

**(A)**

**
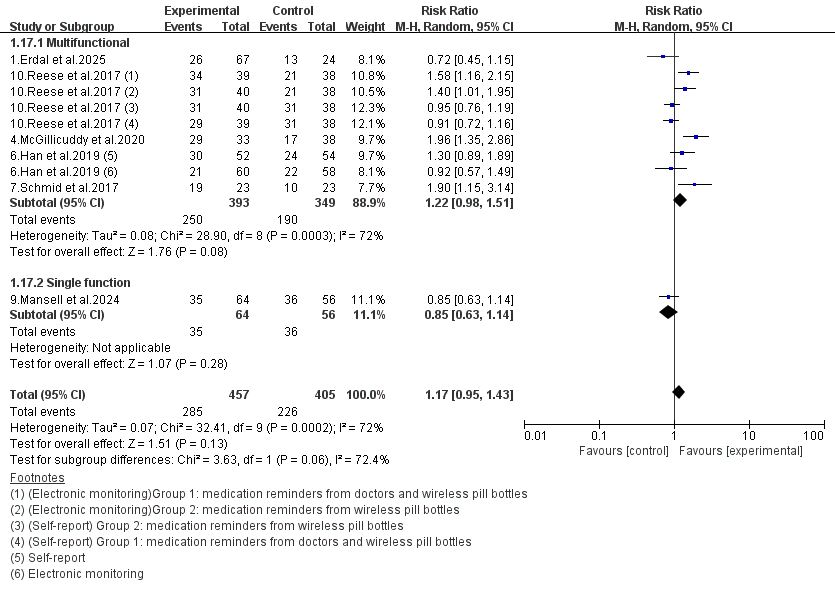
**

**(B)**


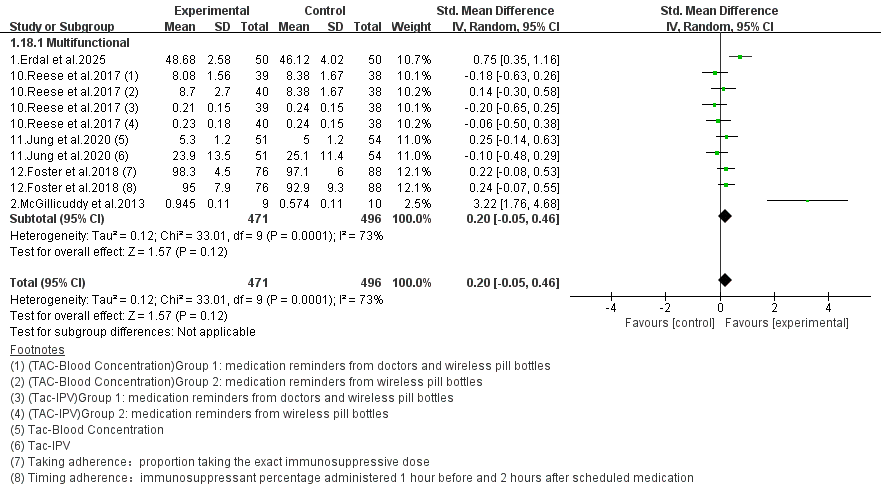


**Fig. S5 Subgroup by ehealth function: (A) dichotomized data, (B) continuous data.**

**
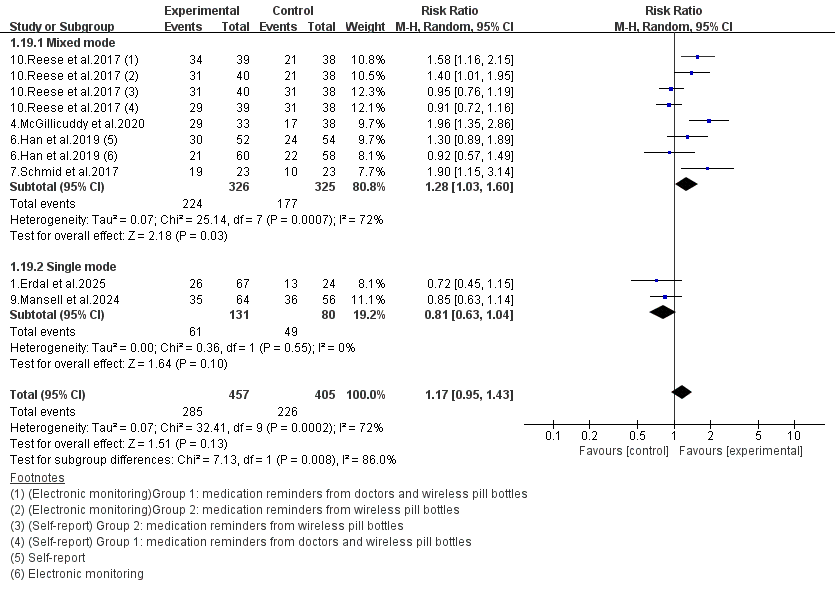
(A)**

**(B)**


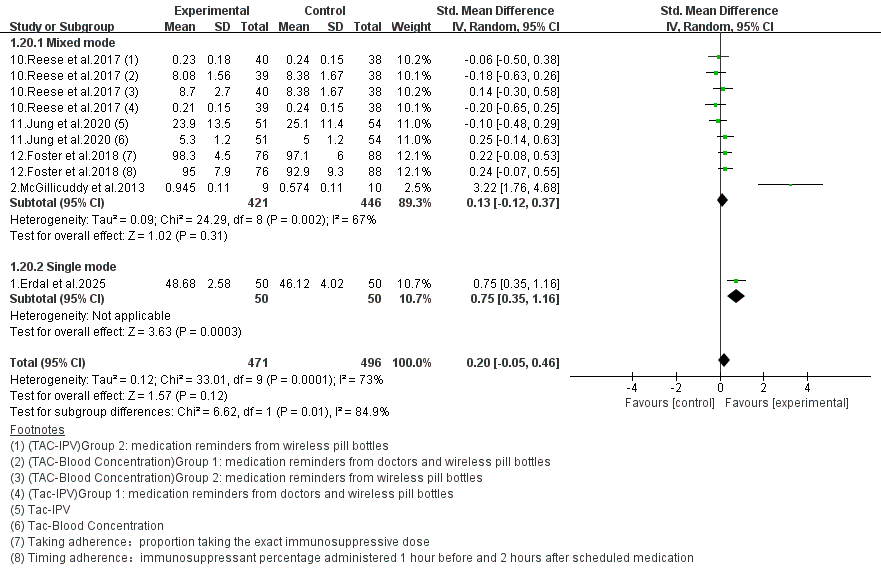


**Fig. S6 Subgroup by ehealth delivery: (A) dichotomized data, (B) continuous data.**

**(A)**


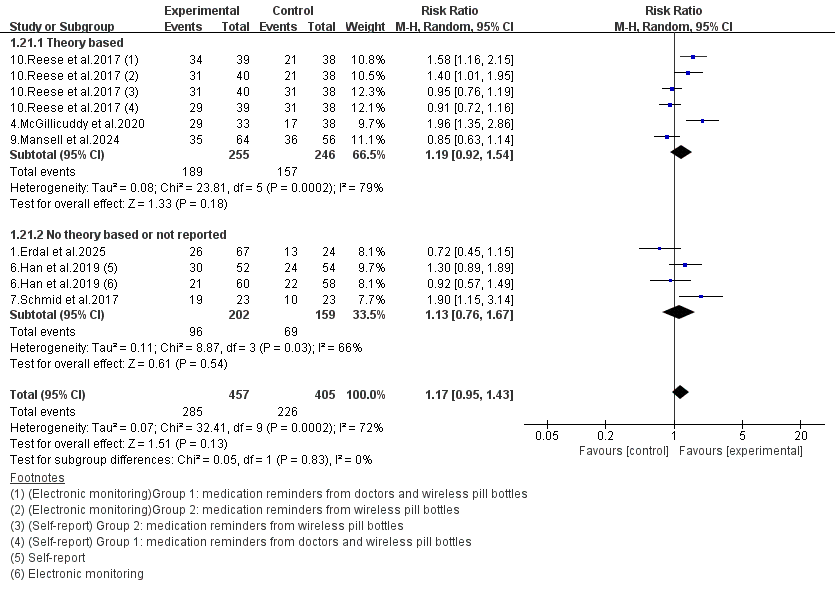


**(B)**


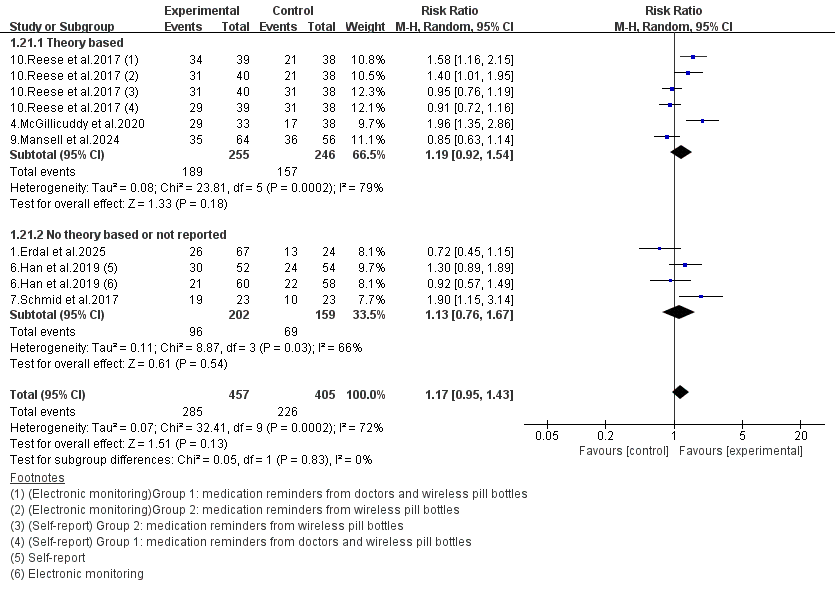
**Fig. S7 Subgroup by theory based intervention: (A) dichotomized data, (B) continuous data.**

**(A)**


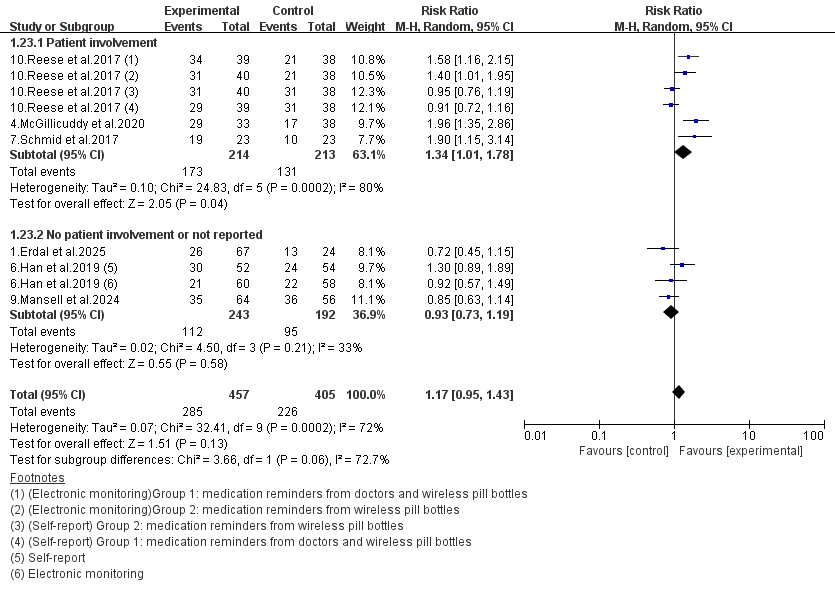


**(B)**


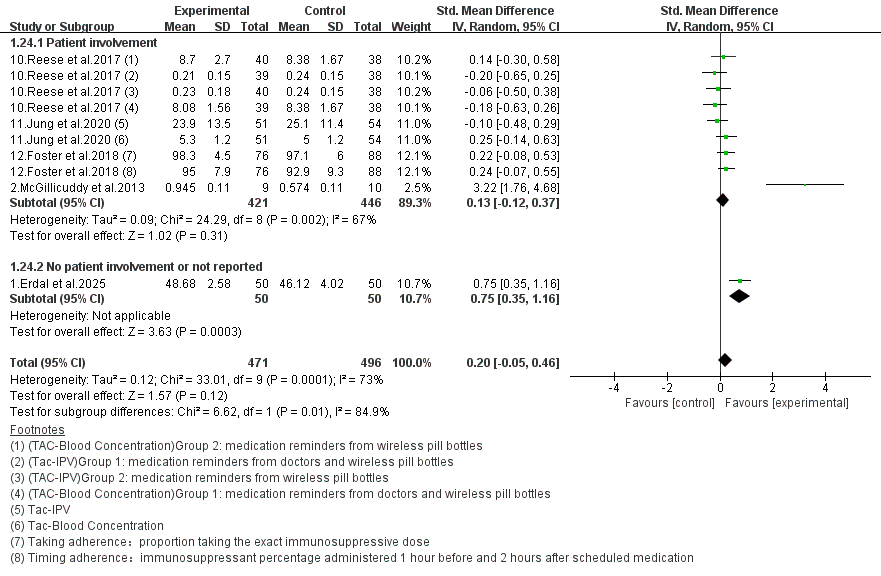


**Fig. S8 Subgroup by user centered design: (A) dichotomized data, (B) continuous data.**
